# Submicron-sized ultrasound contrast agents as therapeutic peptide delivery vehicles in type 1 diabetes

**DOI:** 10.7150/thno.126925

**Published:** 2026-05-11

**Authors:** Mark Ciccaglione, Eric Abenojar, Theresa Kosmides, James E. DiLisio, Kristen A. McDaniel, Kathryn Hartmoore, Anne Gresch, Dillon K. Jarrell, David G. Ramirez, Maura Sticco-Ivins, Aaron W. Michels, Kathryn Haskins, Agata A. Exner, Richard K.P. Benninger

**Affiliations:** 1 Department of Bioengineering, University of Colorado Anschutz Medical Campus, Aurora, CO, USA.; 2 Department of Radiology, Cleveland, OH, USA.; 3 Department of Biomedical Engineering Case Western Reserve University, Cleveland, OH, USA.; 4 Department of Immunology & Microbiology, University of Colorado Anschutz Medical Campus, Aurora, CO, USA.; 5 Barbara Davis Center for Diabetes, University of Colorado Anschutz Medical Campus, Aurora, CO, USA.; 6 Department of Pediatrics, University of Colorado Anschutz Medical Campus, Aurora, CO, USA.; 7 Department of Medicine, University of Colorado Anschutz Medical Campus, Aurora, CO, USA.

**Keywords:** ultrasound contrast agents, peptide delivery, type 1 diabetes, autoimmunity, inflammation

## Abstract

**Background:**

Type 1 diabetes (T1D) is an autoimmune disease where autoreactive T cells infiltrate pancreatic islets, resulting in beta-cell destruction. Antigen-specific immunotherapy with tolerogenic peptides to induce peripheral tolerance has shown promise in preclinical studies but has not shown clinical efficacy.

**Methods:**

Here, we develop peptide-nanobubbles (NBs) as an image-guided platform for induction of peripheral immune tolerance in mouse models of T1D. Sub-micron sized NB ultrasound contrast agents can passively accumulate in pancreatic islets of non-obese diabetic (NOD) mice during the development of diabetes as a result of increased microvascular permeability. We incorporated an insulin B:9-23 peptide mimotope into NBs to target peptides to pancreatic islets for expansion of islet-resident regulatory T cells.

**Results:**

NBs retained normal size distribution and acoustic properties following peptide incorporation. Peptide-NBs accumulated in islets of NOD mice and this accumulation could be visualized in real time using contrast enhanced ultrasound. This resulted in an increased proportion of islet insulin-reactive regulatory T cells. Further, peptide-NBs prepared with a hybrid insulin peptide (HIP) expanded islet HIP-reactive regulatory T cells and substantially delayed diabetes onset in an adoptive transfer mouse model of autoimmune diabetes.

**Conclusions:**

Peptide-NBs offer a promising ‘theranostic’ approach for induction of peripheral tolerance in T1D while monitoring delivery and action via ultrasound contrast.

## Introduction

Autoimmune diseases are conditions in which healthy tissues are attacked by the adaptive immune system. Approximately 10% of Americans are affected by at least one autoimmune disorder, with incidences continuously rising 1, 2. However, most treatments for autoimmunity act by inhibiting functions of adaptive immunity systemically without specificity towards the self-antigens that cause autoimmunity or towards the sites of diseas*e*. This results in patients being at a substantially greater risk for infections and undesirable or dangerous side effects. Type 1 Diabetes (T1D) is an organ specific autoimmune disease characterized by rapid and severe onset of hyperglycemia resulting from infiltration of autoreactive T cells into pancreatic islets and subsequent destruction of insulin-producing beta-cells 3-5. Onset of hyperglycemia is often predated by a “presymptomatic” phase (also known as stages 1 and 2 of T1D), where autoimmunity and immune infiltration have begun, but there is still sufficient beta-cell mass to regulate glucose homeostasis. While it is typical to observe insulitis and some level of dysglycemia (stage 2), this stage of disease progression does not include severe, overt hyperglycemia, which is usually the precipitating event leading to a clinical diagnosis of T1D. 6. In order to preserve beta-cell mass, it is crucial to both diagnose and treat T1D during this presymptomatic phase, which generally occurs several years before the clinical onset of diabetes 5. The only therapeutic that has been clinically approved for presymptomatic T1D is Teplizumab (e.g., an anti-CD3 monoclonal antibody), which has been shown to delay diabetes onset, with patients being identified as positive for presymptomatic T1D through the measurement of circulating islet autoantibodies and impaired glucose tolerance 7-10. However, it does not provide specificity of action, modulating the function of all T cells regardless of autoreactivity and location, and is not effective in preventing diabetes in many patients. Incorporating anti-CD3 antibodies into nanoparticles targeted to pancreatic tissue in preclinical models of T1D has been shown to significantly improve the ability of these antibodies to delay diabetes onset 11. However, this approach is still not antigen-specific, thereby increasing risk for off-target immunosuppression.

A promising approach to address the lack of specificity with broadly acting therapeutics in autoimmune diseases is antigen-specific immunotherapy. Insulin B chain peptides are well-established autoantigens in both non-obese-diabetic (NOD) mice and in human T1D 12-14. Exposure to exogenous antigenic peptides can provide tolerance towards these antigens through the expansion of anti-inflammatory regulatory T cells, thereby preventing autoimmunity against these antigens and onset of diabetes 15-17. While this approach has been promising in mouse studies, there has been limited success in successfully translating antigen-specific immunotherapy to the clinic, with oral insulin and nasal insulin trials showing little to no effect on the prevention of T1D in humans 18, 19.

A method that may improve the efficacy of tolerogenic peptide therapy is incorporation with nanoparticles for more controlled and favorable therapeutic delivery 20-25. For instance, treatment of mice that had received an adoptive transfer of diabetogenic BDC2.5-TCR T cells with PLGA nanoparticles containing 2.5HIP peptide substantially enhanced the ability for the peptide to expand BDC2.5-TCR regulatory T cells and prevent onset of diabetes, perhaps due to favorable uptake and presentation of antigens by antigen-presenting cells for induction of tolerance 20. However, major gaps remain in the inability both to assess therapeutic efficacy and to visualize therapeutic delivery and localization in real time. ‘Theranostic’ approaches and technologies combine diagnostic elements with therapeutics, such as delivery vehicles that can be visualized and modulated in real-time with medical imaging. Utilizing theranostic approaches could be greatly beneficial in enhancing efficacy and functionality of therapeutics to prevent and delay T1D onset.

One such theranostic approach is the utilization of lipid bubbles as both therapeutic delivery vehicles and ultrasound contrast agents 26-31. Ultrasound modalities are extremely safe with no ionizing radiation, cost effective, easily deployable, and portable, and feature high spatial and temporal resolution 32-34. Contrast-enhanced ultrasound uses gas filled microbubbles and non-linear signal detection and is clinically approved for several indications. Nanobubbles (NBs) are sub-micron sized bubbles (200-300 nm in diameter) consisting of a phospholipid shell and heavy gas core that can be visualized, distinguished from tissue, and burst noninvasively with contrast-enhanced ultrasound (CEUS) 31. NBs extravasate into peripheral tissue as a result of elevated microvascular permeability, including inflammatory sites such as those that occur in islets of mice and humans developing T1D 35-38. NBs injected intravenously in NOD mice accumulated in the pancreas but not kidney or pancreas of control NOD-SCID mice, and this accumulation could be visualized using CEUS 39. Given their disease-specific accumulation in inflamed tissue, such as islets in T1D, as well as their ability to be visualized and burst with CEUS, NBs hold promise as image-guided therapeutic delivery vehicles for T1D. However, NBs have only been investigated for the delivery of small-molecule payloads in the context of cancer 26-30, and not peptide-based therapeutics, nor in the context of inflammation or autoimmunity.

In this study we sought to apply nanobubble ultrasound contrast agents as islet-targeted, image-guided peptide delivery vehicles, where nanobubbles accumulate in pancreatic islets and deliver the peptide that is incorporated in the bubble. We utilized an insulin B:9-23 mimotope peptide and a hybrid-insulin peptide (HIP) to determine if peptide-loaded nanobubbles are effective at expanding antigen-specific regulatory T cells in islets to modify the progression of diabetes in mouse models of T1D. Peptides could be incorporated in the nanobubble lipid shell without affecting nanobubble or biologic peptide functions. Upon IV delivery, these peptide-nanobubbles accumulated in islets of NOD mice and expanded insulin-reactive or HIP-reactive regulatory T cells specifically in islets. Importantly, administration of insulin peptide-nanobubbles slightly delayed diabetes onset in NOD mice and treatment with 2.5HIP-nanobubbles substantially delayed the onset of diabetes in an adoptive transfer model of autoimmune diabetes.

## Materials and Methods

### Animals

All animal procedures were performed in accordance with protocols (#00024) approved by the Institutional Animal Care and Use Committee of the University of Colorado Anschutz Medical campus. Female NOD mice and female NOD-SCID mice were purchased from Jackson Laboratories (Bar Harbor, ME) at age 6-8 w. and treated and/or imaged at age 9-10 w. Female and male NOD Foxp3^-^EGFP mice were purchased from Jackson Laboratories at age 6 w. or bred in house and female GFP^+^ mice were treated at age 9 w. Female NOD-BDC2.5TCR-Tg mice were purchased from Jackson Laboratories at age 6-8 w. Throughout the study, animals were monitored weekly for blood glucose concentration utilizing a Bayer Contour or Contour Next blood glucometer.

### Peptides

Non-labeled and FITC-labeled insulin B:9-23 peptide mimotope (C19S, R22E) with 6-residue linker (KKGGCG-SHLVEALYLVSGEEG) was purchased from Biomatik (Kitchener, Ontario, Canada). Palmitoylated non-labeled insulin B:9-23 peptide (Pal-KKGGCG-SHLVEALYLVSGEEG), HIP1 peptide (Pal-RGG-LQTLALWSRMD-GGR), HIP2 peptide (Pal-KKGGCG-LQTLALWSRMD), soluble HIP (GGR-LQTLALWSRMD-RGG), and HEL (irrelevant control) peptide (Pal-KKGGCG-AMKRHGLDNYRGYSL) were purchased from Genemed Synthesis (San Antonio, TX). Palmitoylated FITC-conjugated insulin B:9-23 peptide was purchased from Biomatik. Palmitoylated FITC-conjugated HIP1 AND HIP2 peptide were purchased from Genemed.

### Nanobubble (NB) synthesis and characterization

A lipid solution was first prepared by dissolving 6.1 mg of DBPC, 2 mg of DPPE, 1 mg of DPPA (Avanti Polar Lipids, Pelham, AL), and 1 mg of mPEG(2000)-DSPE (Laysan Lipids, Arab, AL) in 0.1 mL of propylene glycol by repeated sonication and heating at 80 °C. For peptide-NBs, peptide was incorporated in the lipid mixture. A second mixture containing 0.8 mL of phosphate buffered saline (PBS) (pH 7.4) and 0.1 mL of glycerol was brought to 80 °C, added to the lipid solution, and then sonicated at room temperature for 10 min. Note that we usually prepared 5-10 batches at a time, adjusting the amounts of reagents accordingly. This solution (1 mL; 9.18 mg/mL lipids) was then transferred to a 3 mL headspace vial and capped with a rubber stopper. The gas inside the vial was then replaced with octafluoropropane gas (AirGas, Cleveland, OH). To activate the bubbles, the vial was then agitated using mechanical agitation for 45 s. Nanobubbles were then isolated from the bubble population based on their buoyancy by centrifugation at 50 rcf for 5 min, after which bubbles greater than 700 nm are expected to rise 0.5 cm or greater. Nanobubble size, concentration, and buoyancy were measured via resonant mass measurement (RMM) (Archimedes, Malvern Panalytical Inc., Westborough, MA). Size distribution was also assessed with dynamic light scattering (Litesizer 500, Anton Paar, Graz, Austria). *In vitro* echogenicity was determined by placing bubble solutions in a custom-made 1.5% (w/v) agarose mold with a thin channel (L x W x H = 22 x 1 x 10 mm). The agarose phantom was fixed over a 12 MHz linear array transducer and imaged using a clinical ultrasound scanner (AplioXG SSA-790A, Toshiba Medical Imaging Systems, Otawara-Shi, Japan). System acquisition parameters were set to contrast harmonic imaging (CHI) with 12.0 MHz harmonic frequency, 0.29 mechanical index (MI), 65 dB dynamic range, and 70 dB gain. The ultrasound signal for each concentration was determined using the preloaded quantification software (CHI-Q). Enhancement by nanobubbles was calculated by normalizing the nanobubble signal to the signal to a control phosphate buffered saline solution. Bubbles were destroyed using the flash/replenish feature (20 flash cycles, 12 MHz harmonic frequency, 1.52 MI).

To prepare rhodamine-labeled NBs, 50 μL of Liss Rhod PE (1,2-dipalmitoyl-sn-glycero-3-phosphoethanolamine-N-(lissamine rhodamine B sulfonyl)) was added to the lipid mixture and the procedure was carried out as described above. To determine colocalization between FITC-peptide and rhodamine-NBs, labeled peptide-NBs were filtered with a Sephadex G-25 PD-10 desalting column (GE Healthcare, Chicago, IL) and diluted 1:7 in PBS (including dilution from the column). The following equimolar peptide concentrations were used in lipid solutions for FITC-conjugated insulin peptide (palmitoylated), insulin peptide (plain), palm-HIP1, and palm-HIP2: 160 μg/mL, 145.9 μg/mL, 148.5 μg/mL, and 143.4 μg/mL, respectively. NBs were then imaged on an LSM800 confocal microscope (Zeiss, Oberkochen, Germany) using a x63 1.2 NA objective and pinhole settings to provide 1 μm z-section thickness. Colocalization was calculated in MATLAB (Mathworks, Natick, MA) as percentage of FITC-positive pixels (pixels with fluorescent intensity greater than a manually-set threshold) amongst rhodamine-positive pixels. Brightfield images of NBs were concurrently collected to confirm presence of intact NBs.

Insulin peptide-conjugated NBs were prepared by adding DSPE-PEG-Peptide to the initial lipid solution in the method detailed above. DSPE-PEG-Peptide was prepared by mixing insulin B:9-23 (C19S, R22E) with 6-residue linker and no palmitoylation (KKGGCG-SHLVEALYLVSGEEG) with DSPE-PEG-MAL (Laysan Bio, Arab, AL) at a 1:2 ratio in PBS at pH 8.0. The solution was then vortexed, reacted on a vial rotator for 4 h at 4 °C, lyophilized, and dissolved in PBS.

### Peptide-nanobubble treatment

Peptide-NBs were prepared as described and diluted 1:5 in PBS. A custom-made 27 G ½″ winged infusion set (Terumo BCT, Lakewood, CO) was attached to a section of polyethylene tubing (0.61 OD × 0.28 ID; PE-10, Warner Instruments, Holliston, MA) and was inserted in the lateral tail vein. NBs were injected as a single bolus of 200 μL (367 μg lipids at 1.83 mg/mL; 7.84 x 10^9^ NBs at 3.92 x 10^10^ NBs/mL). An equivalent total dosage of peptide was administered as a positive control, and an equivalent number of blank NBs or saline were administered as negative controls. For experiments tracking changes in pancreatic NB accumulation in response to insulin peptide therapy, a total dosage of 14 μg of palmitoylated insulin peptide was administered intravenously over the course of five injections over 2 w. For dexamethasone and insulin peptide-nanobubble co-administration, mice were injected 50 μg dexamethasone I.P. daily for two days prior to the first NB injection and then daily 2 h before each NB injection for 5 consecutive days. Nanobubbles were formed from a lipid precursor solution on the day they were used. For experiments in which mice were treated daily, nanobubbles were prepared separately every day. Nanobubbles were injected in mice within 2-3 h of being prepared.

### Histology and insulitis morphology

For assessment of NB extravasation and insulitis, all animals were anesthetized by I.P. injection of ketamine (80 mg/kg) and xylazine (16 mg/kg) until no longer reactive to toe pinch. The pancreas, spleen, kidney, intestine, and liver were dissected, and mice were euthanized by exsanguination and/or bilateral thoracotomy. Tissues were fixed in paraformaldehyde at 4 °C overnight, cryoprotected in 30% sucrose overnight, embedded in OCT, and stored at -80 °C. 12 μm (peptide-NB coverage) or 8 μm (insulitis) sections were obtained. For assessment of insulitis, sections were stained by Hematoxylin and Eosin (H&E). Images were acquired on an Eclipse-Ti wide field microscope (Nikon, Tokyo, Japan) with a 20 × 0.75 NA Plan Apo objective with a color CCD camera. Images of islets were scored based on the extent of infiltration/insulitis: grade 0, no insulitis; grade 1, peri-insulitis with immune infiltrate bordering; grade 2, immune infiltrate penetrating the islet, covering <50% of the islet area; and grade 3, immune infiltrate penetrating the islet, covering >50% of the islet area. Weighted averages were calculated for each animal. For assessment of NB extravasation, 10 w female NOD or NOD-SCID mice were treated with a single bolus injection of peptide-NBs (from a lipid solution containing 350 μg/mL palm-Ins) as described, with peptide labeled with FITC and NBs labeled with rhodamine. Following 30 min for NB extravasation, mice were anesthetized, and abdominal tissues were isolated and processed as described. Sections were stained with DAPI and imaged on an LSM800 confocal microscope (Zeiss) using a x20 0.8 NA objective and pinhole settings to provide 1 μm z-section thickness throughout the tissue depth. Sections were also imaged on an Olympus VS200 slide scanner using a x20 objective. Rhodamine and FITC coverage were calculated in MATLAB as the percentage of rhodamine or FITC positive pixels (pixels with fluorescence intensity >2 standard deviations above the background fluorescence intensity) across the region of interest.

### Contrast enhanced ultrasound (CEUS) imaging

General anesthesia was established with isoflurane inhalation for a total of 40–50 min, abdominal fur was removed using depilatory cream, and ultrasound coupling gel placed between the skin and transducer. Foot pad electrodes on the ultrasound machine platform monitored the animal’s electrocardiogram and respiration rate and a mouse rectal probe monitored body temperature. All animals were constantly monitored throughout the imaging session to maintain body temperature and respiration rate. A VEVO 2100 small animal high-frequency ultrasound machine (Visual Sonics, Fujifilm, Toronto, Canada) was used for all experiments. Data collection used Vevo Lab Version 5.5.0. For CEUS imaging a MS250 linear array transducer was used at a frequency of 18 MHz. B-mode imaging (transmit power 100%) was performed prior to NB injection to identify anatomy of the pancreas body, based on striated texture and location in relation to the spleen and kidney. Following identification of the pancreas and selection of a region of interest, sub-harmonic contrast mode was initiated. Acquisition settings were set at: transmit power 10% (MI=0.12), frequency 18 MHz, standard beamwidth, contrast gain of 30 dB, 2D gain of 18 dB, with an acquisition rate of 26 frames per second. Gating to remove movements as a result of animal breathing was carried out in MATLAB. NBs were diluted 1:5 in PBS and injected as a single bolus of 200 μl solution (from a lipid solution containing 350 μg/mL palm-Ins). Background data were acquired for 3 min prior to injection. Time courses were stored at 30s intervals up to a 30 min duration post-injection. For analysis of NB contrast, regions of interest for the pancreas and kidney were identified by the B-mode image based upon anatomical features and texture in VevoCQ (Vevo Lab Version 5.5.0). Data were exported into MATLAB, where frames during respiration were removed and the NB signal was background subtracted by the averaged contrast intensity taken before injection.

### *In vitro* T cell receptor stimulation

For stimulation of PCR1-10 cells (insulin-reactive T cell hybridoma), nanobubbles and peptide-NBs (from a lipid solution containing 70 μg/mL peptide) were prepared, filtered with a Sephadex G-25 PD-10 desalting column (GE Healthcare) and diluted 1:7 in PBS (including dilution from column). Peptide controls were prepared in PBS at an equivalent concentration. A single cell suspension was obtained from the spleen of a non-diabetic female NOD mouse as a source of antigen presenting cells. Following red blood cell lysis, 200,000 splenocytes suspended in 100 μL of complete media were combined with 150 μL of the treatment. For all experiments, complete media consists of 10% FBS, 0.68 mg/mL dextrose, 0.30 mg/mL glutamine, 0.68% 50X essential amino acids (Gibco), 1.27% 100X non-essential amino acids (Gibco), 0.1 mg/mL sodium pyruvate, 0.77 mg/mL sodium bicarbonate, 45 mg/L gentamycin, 55 mg/L penicillin G, 91 mg/L streptomycin sulfate, and 44 mM 2-ME). 200,000 PCR1-10 T cells were then suspended in 50 μL of complete media, added to the mixture, and cultured overnight at 5% CO2/37 °C. Cells were then spun and the supernatant was analyzed via ELISA for IL-2. *Ex vivo* stimulation of CD4^+^ NOD-BDC2.5TCR-Tg T cells utilized the same protocol, except CD4^+^ cells were magnetically sorted from splenocytes obtained from a NOD-BDC2.5TCR-Tg mouse and used in place of a T cell hybridoma. NBs were made from a lipid solution containing 289 μg/mL palm-HIP2 or 376 μg/mL palm-HEL and filtered with a Sephadex G-25 PD MiniTrap column (Cytiva).

### Flow cytometry

For assessment of insulin-reactive regulatory T cells in NOD mice, NOD Foxp3-EGFP mice were treated as described at 9 w, where peptide-NBs were made from a lipid solution containing 350 μg/mL palmitoylated insulin peptide and HEL-NBs were made from a lipid solution containing 376 μg/mL palm-HEL peptide. At 12 w mice were anesthetized by I.P. injection of ketamine (80 mg/kg) and xylazine (16 mg/kg) until no longer reactive to toe pinch. Spleens, pancreatic lymph nodes, and islets were harvested, and single cell suspensions were obtained for spleen and lymph nodes. Islets were cultured overnight at 5% CO2/37 °C in complete media. Following red blood cell lysis for spleens, cells were washed and blocked with anti-CD16/anti-CD32 (Fc block) at 1 μg/mL at 4 °C. Following Fc Block and washing, cells were incubated with two insulin-I-A(g7) tetramers (p8E and p9E) obtained from the NIH Tetramer Core Facility at Emory University (Atlanta, GA) at 20 μg/mL each and Anti-TCRβ (HAM67) at 10 μg/mL in a 60/40 mixture of media and FACS buffer (PBS, 1% FBS, 15 mM sodium azide) at 37 °C for 2 h and were checked every 30 min to avoid cell sedimentation. Cells were washed and stained for 30 min at 4 °C for each of the following at a concentration of 0.2-0.25 μg/100 μL: CD11b, CD11c, and F4/80 PerCP-Cy5.5 (dump), CD4 APC, CD8 BV650, CD25 APC-Cy7, CD45R (B220) AF700, CD44 PE-Cy7, CD62L eFluor 450, CD45 BV711, LAG-3 BV785, CD49b PE/Dazzle 594 and Zombie Aqua viability stain (Biolegend). To validate tetramers, AS91, 5KC, and 3BK T cell hybridomas were cultured in complete media with 10% FBS at 5% CO_2_/37 °C. Cells were maintained at a concentration of 0.2-0.4x10^6^ cells/mL. Cells were stained with either PE-labeled Anti-TCRβ at 10 μg/mL or tetramers as described. All antibodies were purchased from Biolegend (San Diego, CA) or Fisher Scientific (Waltham, MA). Cells were analyzed with an Cytek Aurora 5-Laser Cytometer (Fremont, CA). For assessment of BDC2.5 HIP-reactive T cells, cells were stained with a 2.5-HIP I-A(g7) tetramer obtained from the NIH Tetramer Core Facility at Emory University (Atlanta, GA). For intracellular Foxp3 staining (used for the BDC2.5 adoptive transfer mice), cells were fixed and permeabilized using a transcription factor staining kit (eBioscience, Invitrogen) and stained with a Foxp3 AF488 antibody (Bioelegend) at 0.25 μg/100 μL. For BDC2.5 adoptive transfer mice, islets were dissociated immediately following isolation by suspending in an enzyme-free dissociation buffer (Gibco) for 30 min at 37 °C. For NOD mice (non-Foxp3GFP) treated with dexamethasone and insulin peptide-nanobubbles, the same protocol was used as analysis of 2.5HIP-reactive T cells but with the insulin-I-A(g7) tetramers (p8E and p9E) described previously.

### Adoptive transfer of BDC2.5 T cells and 2.5HIP NB treatment

For adoptive transfer of BDC2.5 T cells, the spleen and lymph nodes (superficial cervical, axillary, pancreatic, and inguinal) from two, 6-8 w old NOD-BDC2.5TCR-Tg mice were strained through a 70 μm filter. Resulting cells were magnetically sorted via negative selection to obtain CD3^+^ cells with a mouse CD3 T cell isolation kit (Biolegend). Cells were resuspended in media at 0.75 million cells/mL and mouse T cell activating (CD3/CD28) Dynabeads (Gibco) were added at a 1:2 bead:cell ratio. Cells were then cultured for 3 days with rIL-2 (2 ng/mL) and rIL-7 (5 ng/mL) at 5% CO_2_/37 °C. Following T cell activation in culture, cells were removed from beads, and 2.5 million activated T cells were transferred via retroorbital injection per NOD-SCID mouse (day 0). On days 1-4, mice received daily treatments of 2.5HIP-NBs (palm-HIP2) via tail vein injection for a total dose of 18 μg per day (72 μg total). Control mice (HEL-NB and peptide only) received an equimolar total dosage of their respective peptides. Peripheral blood glucose was monitored twice per week, and animals were considered diabetics after two consecutive readings above 250 mg/dL.

### Measurement of peptide encapsulation and release

To quantify the encapsulation efficiency (EE) of FITC-palmitoylated insulin peptide in bubbles, unentrapped peptide was removed from the isolated NB fraction by differential centrifugation at 700 g for 10 min. The infranatant (‘free’ unentrapped peptide) was collected and the remaining supernatant (bubbles) was resuspended in 1 mL PBS. To quantify entrapped peptide, the bubbles were heat sonicated at 50 °C for 20 min to release entrapped peptide. FITC fluorescence from free unentrapped peptide and bubble-entrapped peptide fractions were quantified using a fluorescence plate reader (TECAN, Infinite M200, Tecan Group Ltd., Switzerland) at Ex/Em 495/520 nm. The entrapment efficiency was quantified from a calibration curve with linearity in the range of 0 – 160 μg/mL and a correlation coefficient greater than 0.99. All samples were measured in triplicates and reported as mean ± standard deviation. The %EE of the peptide in bubbles was calculated using the following equation:



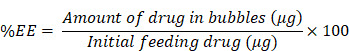



To evaluate the release of the peptide from NBs, isolated FITC-labeled palmitoylated insulin peptide-NBs were resuspended in 1 mL PBS (pH 7.4). The sealed vial was placed at 37 °C with constant shaking at 80 rpm. At designated time points (t = 0, 0.5, 1, 2, 6 h), the vial was inverted and centrifuged at 700 g for 10 min. Infranatant (100 – 200 μL) was collected and replaced with equivalent volumes of PBS to facilitate sink conditions. The infranatant was then centrifuged at 21,300 g for 4 min to collect the released fractions of the peptide. The released peptide was quantified using a fluorescence plate reader with Ex/Em 495/520 nm.

### Statistics and reproducibility

All error bars represent standard error of the mean. Comparisons between experimental groups utilized a two-tailed t test in the case of comparing two groups, or ANOVA in the case of comparing multiple groups. Survival Analyses utilized a log-rank test. Correlations between two variables utilized simple linear regression.

## Results

### Insulin peptide palmitoylation enables incorporation in lipid nanobubbles without affecting nanobubble properties

**Figure 1:**
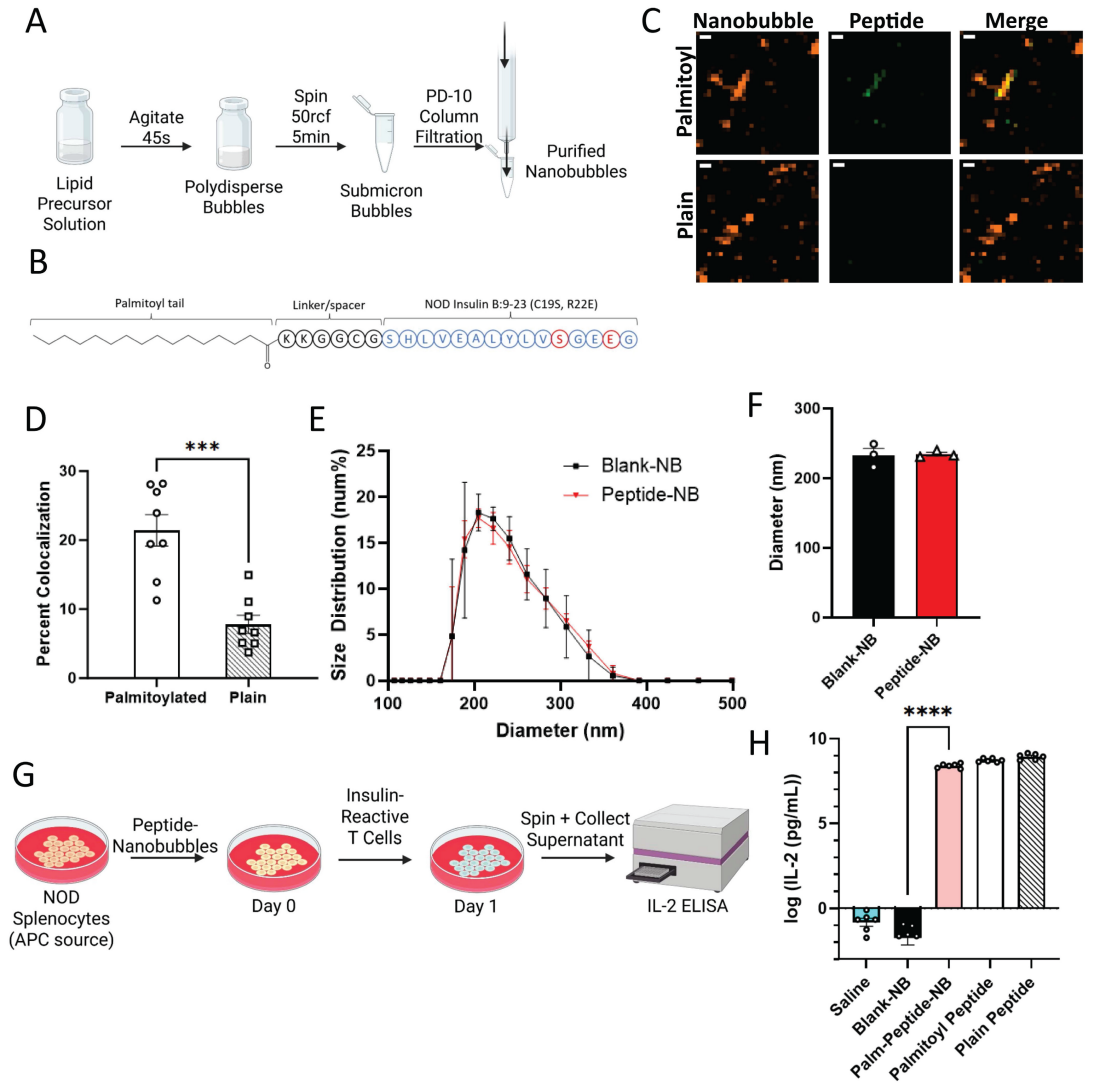
** Peptide palmitoylation enhances incorporation in nanobubbles without affecting size distribution or peptide bioactivity. A**. Schematic illustrating process of forming nanobubbles from a precursor lipid solution. **B** Schematic illustrating palmitoylated insulin peptide design. **C** Representative confocal images of rhodamine-nanobubbles (orange) containing FITC-labeled palmitoylated peptide (green; top) or non-palmitoylated “plain” peptide (green; bottom). **D** Mean percent colocalization of rhodamine-nanobubbles and FITC-peptide between palmitoylated and plain insulin peptide. **E** Nanobubble size distribution with and without peptide inclusion. **F** Mean diameter of nanobubbles in (**e**). **G** Schematic illustrating experimental process for evaluation of peptide function. **H** Effect of nanobubble-inclusion and peptide palmitoylation on peptide ability to elicit T cell response. Error bars in (**D-F, G**) represent s.e.m. Data in (**D**) represent n = 8 lipid solutions. Data in (**E-F**) represent n = 3 lipid solutions. Data in (**H**) represent n = 6 lipid solutions. Scale bars in (**C**) represent 500 nm. ***p < 0.001, ****p < 0.0001. (**D**) p = 0.0001, comparing groups indicated (t test, two-tailed). (**H**) p < 0.0001, comparing palmitoylated-peptide nanobubbles and blank nanobubbles (ANOVA).

Previous work has demonstrated that “nanobubble (NB)” submicron ultrasound contrast agents accumulate in pancreatic islets of non-obese diabetic (NOD) mice as a result of enhanced microvascular permeability associated with insulitis 39. In order to use nanobubbles to deliver peptides to the islet microenvironment, we incorporated insulin B:9-23 peptide mimotopes in the NB lipid shell. Insulin B:9-23 is a known autoantigen in NOD mice and treatment with an insulin peptide mimotope (Insulin B:9-23 R22E) has been explored as a tolerogenic therapeutic for the prevention of T1D in mice 12, 13, 40-42. We first linked a palmitoyl (C16) fatty acid tail to the N-terminus of the peptide (insulin B:9-23 C19S/R22E) with a 6-residue glycine-rich linker segment to enhance solubility in the NB lipid shell (Figure 1A). NBs were labeled with rhodamine and peptide was labeled with FITC. Following peptide-NB formation and filtration (Figure 1B), peptide-NBs were imaged for colocalization as a measure of efficiency of incorporation. Colocalization was significantly higher for palmitoylated peptide than non-palmitoylated peptide (Figure 1C-D, Figure S1). Therefore, palmitoylated peptide-nanobubbles are used throughout experiments.

Next, we determined if NB physical properties are affected in peptide-NBs. Using dynamic light scattering as well as a resonant mass measurement technique developed in-house, we characterized the effect of peptide inclusion at two different concentrations 38. Inclusion of peptide did not affect NB diameter or concentration (Figure 1E-F, Figure S2A-E). Additionally, peptide inclusion did not lessen the formation of buoyant particles (bubbles) in comparison to non-buoyant particles (Figure S3). Peptide inclusion did, however, slightly increase NB polydispersity (Figure S2F). The magnitude of nanobubble charge decreased very slightly, with a zeta potential of -49.2 mV for blank-NBs and a zeta potential of -45.4 mV for peptide-NBs.

We then assessed the impact of peptide palmitoylation and nanobubble-incorporation on peptide bioactivity. Insulin peptide mimotope function relies on uptake by antigen presenting cells (APCs), presentation to T cells, and interaction with insulin-reactive T cell receptors (TCRs). Therefore, we first assessed if this process is impacted by peptide palmitoylation and/or incorporation in NBs *in vitro*. Using a primary culture of NOD splenocytes as a source of APCs, APCs were pulsed with peptide-NBs and subsequently co-cultured with an insulin-reactive T cell hybridoma (PCR1-10). Since these cells produce IL-2 in response to TCR stimulation by APC-presented peptide, IL-2 production was used as a measure of peptide function (Figure 1G). IL-2 production remained similar with both peptide palmitoylation and incorporation in NBs and was significantly and substantially higher than blank NB or saline controls (Figure 1H). In contrast, covalent conjugation of peptide to NBs eliminated peptide bioactivity, further indicating the benefit of peptide palmitoylation (Figure S4). Therefore, incorporation of palmitoylated peptide via lipophilic attractions with the NB lipid shell was utilized for all experiments rather than covalently conjugated peptide.

We further characterized the encapsulation efficiency and release dynamics of the palmitoylated peptide by separating FITC-labeled free peptide from nanobubble-entrapped peptide by differential centrifugation. We found an average encapsulation efficiency of 81% (Figure S5A). Over the course of 6 h, approximately half of the peptide is released from nanobubbles (Figure S5B). After this point, the bubbles themselves degrade. Therefore, we expect <20% of peptide to be released from nanobubbles following their preparation from the precursor lipid solution, prior to injection.

These results show that insulin peptides can be incorporated in the NB lipid shell, the efficiency of peptide incorporation is enhanced through peptide palmitoylation, and physical NB properties and peptide bioactivity are largely unchanged.

### Nanobubbles target insulin peptide delivery to NOD islets

**Figure 2:**
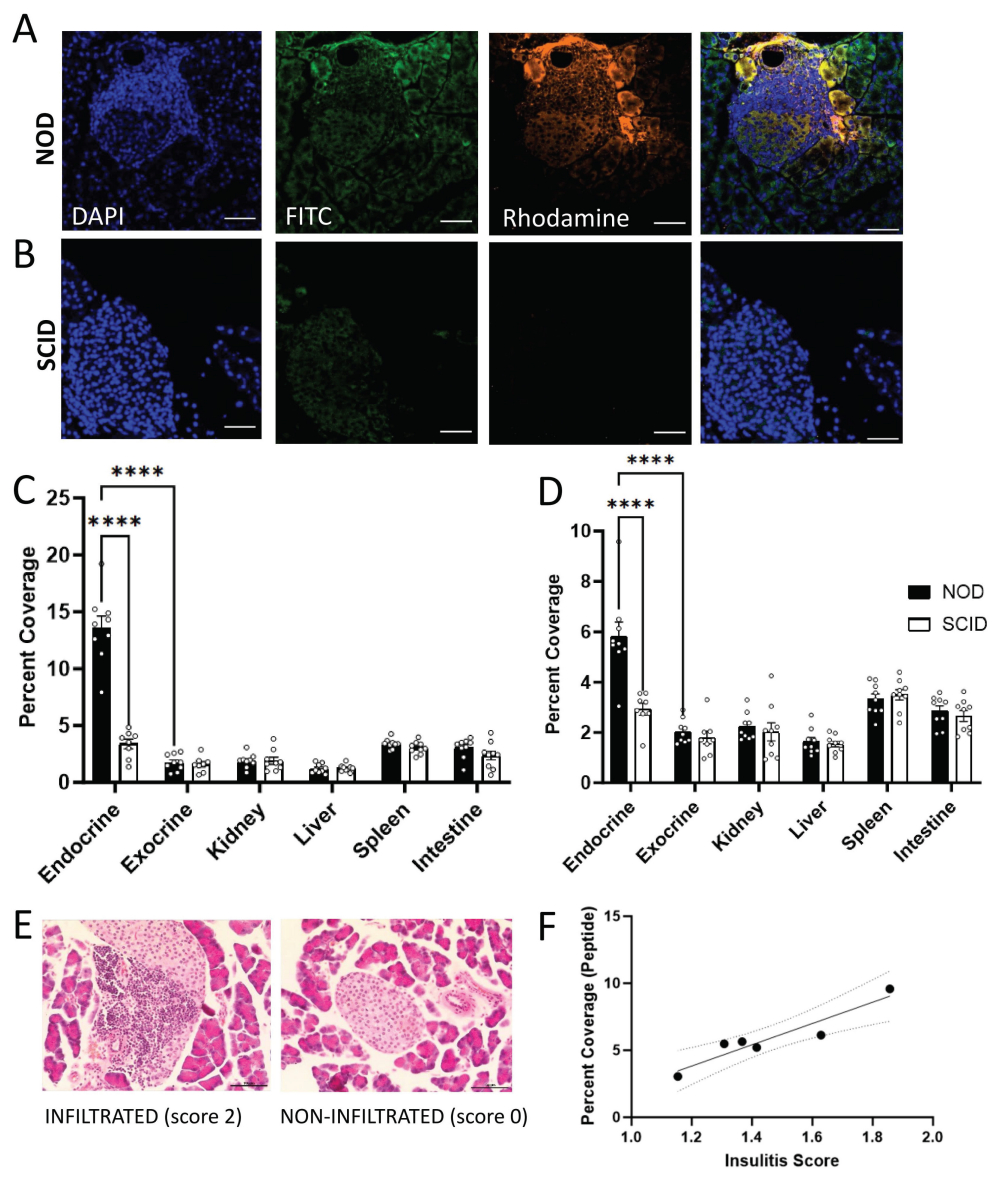
** Nanobubbles can target insulin peptide delivery to islets in non-obese diabetic mice. A** Representative confocal images of an islet within pancreas sections from a 10 w old NOD mouse 30 min post-injection of rhodamine-labeled nanobubbles (orange) containing FITC-labeled palmitoylated insulin peptide (green), counterstained with DAPI (blue). **B** As in (**A**) for a 10 w old SCID mouse. **C** Mean rhodamine coverage in pancreatic islets (endocrine), pancreatic acinar tissue (exocrine), kidney, liver, spleen, and intestine of 10 w old NOD and SCID mice. **D** As in (**C**) for FITC (palmitoylated peptide) coverage. **C** Mean rhodamine coverage in pancreatic islets (endocrine), pancreatic acinar tissue (exocrine), kidney, liver, spleen, and intestine of 10 w old NOD and SCID mice. **D** As in (**C**) for FITC (palmitoylated peptide) coverage. **E** Representative images of hematoxylin and eosin (H&E) stained pancreas sections from a 10 w old NOD mouse featuring an infiltrated islet (left; insulitis score = 2) and a non-infiltrated islet (right; insulitis score = 0). **F** Scatterplot of FITC (peptide) percent coverage vs. insulitis score for 10 w old NOD mice. Error bars in (**C-D**) represent s.e.m. Trend line in (**H**) indicates simple linear regression with 95% confidence intervals. Data in (**C-D**) represent N = 9 NOD and N = 9 SCID mice (8 SCID pancreas measurements). Data in (**F**) represent N = 6 NOD mice. Scale bars in (**A-B, E**) represent 50 μm. ****p < 0.0001. (**C-D**) p < 0.0001 comparing, for both rhodamine and FITC, NOD and SCID endocrine and NOD endocrine and exocrine (ANOVA). (**F**) p = 0.0055 (simple linear regression).

To determine if NBs can passively target peptide delivery to the NOD pancreas, we characterized the accumulation of peptide-NBs in abdominal tissues. NBs and peptide were fluorescently labeled with rhodamine and FITC, respectively, and fluorescently-labeled peptide-NBs were injected intravenously in NOD and NOD-SCID (immunocompromised control) mice. After 30 min, to allow for accumulation of peptide-NBs in peripheral tissue and clearance from vasculature, abdominal organs (pancreas, spleen, kidney, intestine, and liver) were dissected. We performed histological analysis to assess accumulation of peptide-NBs in each of these tissues at 30 min post-injection, distinguishing pancreatic endocrine tissue (islets) from exocrine tissue via DAPI staining and autofluorescence (Figure 2A-B). NBs accumulated in NOD endocrine pancreas tissue (Figure 2A and C, Figure S6A), but not in NOD-SCID endocrine pancreas tissue (Figure 2B-C). Palmitoylated peptide delivered by NBs also accumulated in a similar fashion (Figure 2A-B and D, Figure S6A-B), indicating that palmitoylated peptide in NBs is stable in circulation following intravenous injection and NBs can target peptide delivery to NOD endocrine pancreas tissue. To account for differences in tissue autofluorescence we also quantified the fluorescent intensity of peptide-nanobubbles normalized to that measured in a non-treated mouse. In NOD pancreatic islets, we again find areas of high intensity nanobubble and peptide fluorescent signal surrounded by little to no signal, while in other tissues we either find no signal (e.g. kidney) or diffuse low intensity signal (e.g. spleen). When quantifying the intensity of nanobubble-labeled tissue regions (Figure S7), we again see an accumulation of nanobubbles in NOD endocrine pancreas.

Prior studies have indicated that the level of NB accumulation in NOD islets depends on the level of insulitis 39. To determine if this is true with regard to peptide delivered to islets by NBs, we stained pancreas sections from the same mice with H&E and obtained average insulitis score (Figure 2E). The extent of palmitoylated peptide accumulation in NOD mice significantly correlated with the extent of insulitis (Figure 2F).

These results indicate that NBs can passively target insulin peptides to islets in NOD mice and the extent of accumulation correlates with disease progression. Further, peptide palmitoylation is necessary for stability in the NB shell following intravenous injection.

### Pancreatic accumulation of insulin peptide-nanobubbles can be visualized with ultrasound

**Figure 3:**
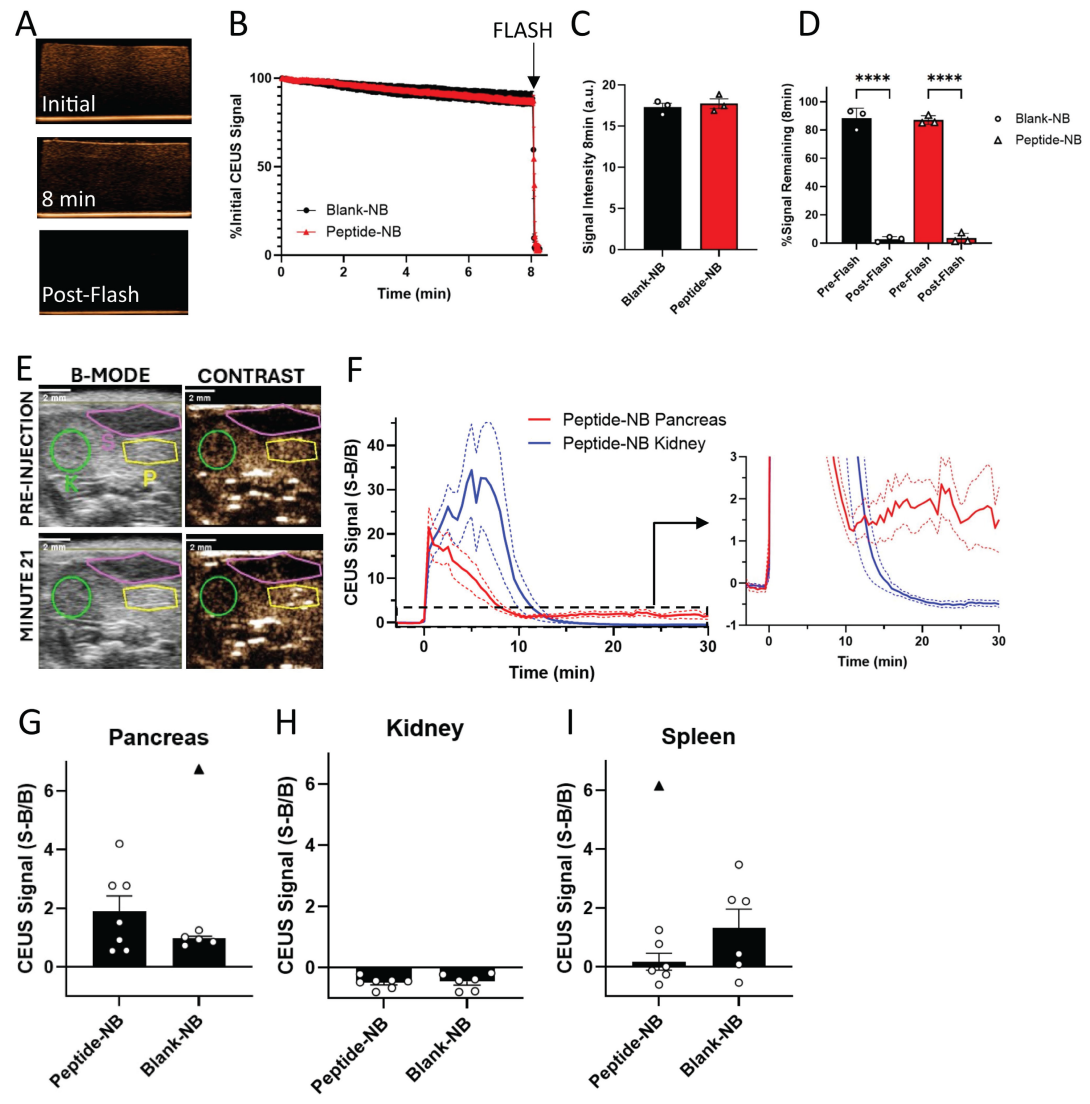
** Nanobubbles’ function as ultrasound contrast agents is not impeded by peptide incorporation. A** Representative ultrasound contrast images of peptide-nanobubbles in a tissue-mimicking phantom immediately following (top) and 8 min following (middle) inclusion of nanobubbles and immediately following nanobubble ablation (“flash”; bottom). **B** Mean time-course of contrast signal of nanobubbles containing no peptide as well as two concentrations of palmitoylated insulin peptide each second for 8 min *in vitro* followed by nanobubble ablation. **C** As in (**B**), mean ultrasound contrast signal intensity at time = 8 min, immediately prior to nanobubble ablation. **D** As in (**B**), mean ultrasound contrast intensity expressed as a percentage of initial intensity at time = 8 min, both prior to and immediately following nanobubble ablation. **E** Representative B-mode (left) and contrast-mode (right) images of a 9 w old NOD mouse pancreas (yellow), kidney (green), and spleen (magenta) before (top) and 21 min after (bottom) injection of peptide-nanobubbles. **F** Mean time-course of contrast signal in the pancreas and kidney of 9 w old NOD mice following peptide-nanobubble injection. Plotted is the Background normalized Signal minus Background, with the baseline itherefore defined as 0. **G** Mean contrast signal in the pancreas averaged between 20-25 min post-injection of peptide nanobubbles in 9 w old NOD mice. **H** As in (**G**) for kidney. **I** As in (**G**), for spleen. Error bars in (**B-D, F-I**) represent s.e.m. Black triangles in (**G-I**) represent outliers (ROUT method, Q = 1%). Data in (**B-D**) represent n = 3 lipid solutions. Data in (**F-I**) represent n = 7 mice. Scale bars in (**E**) represent 2 mm. ****p < 0.0001. (**D**) p < 0.0001 comparing ultrasound contrast signal pre-and-post ablation for all groups indicated (ANOVA).

Our *in vitro* data indicate that basic properties of NBs, including concentration and average diameter, are not substantially affected by peptide (Figure 1). We next further tested the impact of peptide on NB function as ultrasound contrast agents. Peptide-nanobubbles were first imaged in a tissue-mimicking phantom (Figure 3A). We tested whether ultrasound contrast signal intensity, short-term stability of this echogenicity, and the ability to ablate or “burst” with a high mechanical-index ultrasound pulse (“flash”) were negatively affected by peptide (Figure 3B). Ultrasound contrast signal intensity from NBs, as well as the change in this signal over minutes were not affected by peptide incorporation. The ability to acoustically ablate NBs was also not affected, with near-complete elimination of contrast signal following flash (Figure 3C-D).

Having demonstrated that peptide does not influence NB echogenicity *in vitro*, we next determined the impact on echogenicity *in vivo*. We focused on characterizing ultrasound contrast-based assessment of NB accumulation in the NOD pancreas. Prior work has demonstrated that ultrasound contrast can be used to measure NB accumulation in the NOD pancreas and distinguish this signal from that of low-accumulation tissues such as the kidney 39. We injected peptide-nanobubbles in NOD mice and imaged ultrasound contrast in the pancreas and kidney continuously for 30 min, using the B-mode scan to localize pancreas, kidney and spleen (Figure 3E). Following an initial post-injection signal spike, the contrast signal rapidly dropped fully to baseline in the kidney following NB clearance from the blood stream. While the contrast signal dropped in the pancreas, it did not drop fully to baseline, indicating pancreatic NB accumulation (Figure 3F). The average contrast signal 20-25 min post-injection was not significantly different between blank-NBs and peptide-NBs (Figure 3G-I), even when excluding outliers that could drive this effect (Figure 3G and I, black triangles). This indicates that ultrasound contrast measurement of pancreatic NB accumulation in NOD mice is not affected by inclusion of peptide. When scanning separate mice from the same cohort with non-peptide-loaded nanobubbles, we see similar dynamics in the pancreas (Figure S8E).

When looking at the temporal dynamics of peptide-nanobubbles in the pancreas and kidney of NOD mice, we observe an initial spike in ultrasound contrast immediately following injection from circulation in the blood stream (Figure S8A). Within the pancreas, approximately 2.5 min post-injection the signal decays exponentially, reflective of clearance from the blood stream with a half-life of 125 s (Figure S8A and C). Conversely, within the kidney the signal continues to increase until approximately 7.5 min post-injection, where upon the signal decays exponentially with a half-life of 40 s (Figure S8A and D). After 15 min post-injection, all of the signal in the kidney has been depleted, while a significant portion remains in the pancreas that is most prominent at 22.5 min post-injection (Figure S8B).

These results indicate that the utility of NBs as echogenic ultrasound contrast agents, as well as their overall echogenic properties, are not affected by peptide inclusion *in vitro* and *in vivo*, meaning their diagnostic potential is retained in the context of peptide therapeutic delivery.

### Insulin peptide-nanobubble treatment expands islet regulatory T cells in NOD mice

**Figure 4:**
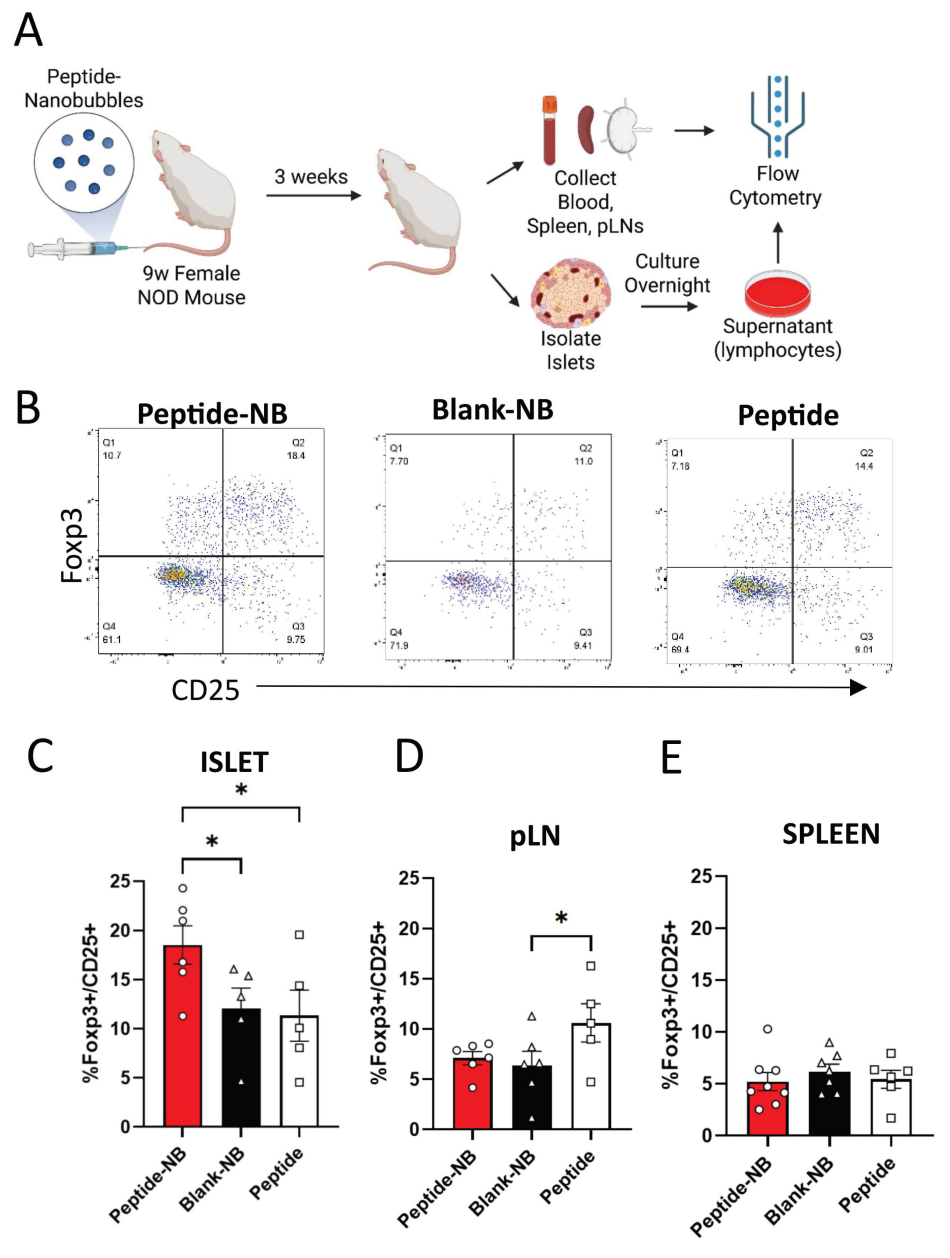
** Insulin peptide-nanobubble treatment expands insulin-reactive regulatory T cells in NOD mouse islets. A** Schematic illustrating experimental plan. **B** Representative flow cytometry gating for the identification of proportion of Foxp3^+^CD25^+^ Tregs amongst insulin-reactive CD4^+^CD8^-^ T cells. **C-E** Proportion of Tregs amongst insulin-reactive CD4^+^ T cells in 9 w old NOD mice that had been treated with peptide-nanobubbles, blank nanobubbles, or peptide in (**C**) islets, (**D**) pancreatic lymph nodes, and (**E**) spleen. Error bars in (**C-E**) represent s.e.m. Data in (**C**) represent n = 6 and n = 5 samples for peptide-NB and blank-NB and peptide, respectively. Data in (**D**) represent n = 6 and n = 5 samples for peptide-NB and blank NB, and peptide, respectively. Data in (**E**) represent n = 8, n = 7, and n = 6 samples for peptide-NB, blank-NB, and peptide, respectively. One sample represents cells from two mice pooled together. *p < 0.05. (**C**) p = 0.0404 comparing peptide-NB and blank-NB, p = 0.0243 comparing peptide-NB and peptide (ANOVA). (**D**) p = 0.0477 comparing blank-NB and peptide (ANOVA).

Given that NBs could deliver insulin peptides to the islets of NOD mice, we examined the therapeutic effect of insulin peptide accumulation in islets following administration of peptide-NBs to NOD mice. Prior studies suggest that insulin peptide mimotopes have potential to expand anti-inflammatory insulin-reactive CD4^+^CD25^+^Foxp3^+^ regulatory T cells (Tregs) 41. We treated 9 w old NOD-Foxp3GFP mice with a single dose of insulin peptide-NBs and then isolated cells from islets, pancreatic lymph nodes (pLNs), and spleen at 12 w of age, and assessed the proportion of insulin-reactive Tregs using flow cytometry (Figure 4A-B). MHC tetramer staining was used to distinguish insulin-reactive T cells. We validated the specificity for the tetramer to specifically label insulin-reactive T cell receptors (Figure S9). The proportion of insulin-reactive tetramer^+^CD4^+^ lymphocytes that are CD25^+^Foxp3^+^ Tregs in islets was greater for peptide-NB-treated mice compared to peptide-treated and blank-NB-treated mice (Figure 4C). Interestingly, this was higher in pLNs for peptide-treated mice rather than peptide-NB-treated mice, potentially reflecting the impacts of systemic administration rather than targeted administration (Figure 4D).

To test if the observed effects on proportion of Tregs in islets were consistent when just examining activated T cells, we assessed differences in proportion of islet Tregs amongst naïve (CD44^low^CD62L^high^) and effector (CD44^high^CD62L^low^) T cells (Figure S10A). We found that the proportion of Tregs amongst effector CD4^+^ insulin-reactive T cells in islets was significantly greater in mice treated with peptide-NBs compared to mice treated with peptide (Figure S10B). We also observed no differences in proportion of islet Tregs amongst non-insulin-reactive CD4^+^ T cells (Figure S10I) and a trend towards a higher islet insulin-reactive Treg:CD8 T cell ratio amongst mice treated with peptide-NBs (Figure S10F). Another T cell population that has garnered interest in the context immune tolerance is CD4^+^LAG3^+^CD49b^+^ T regulatory type 1 (TR1) cells 43, 44. We found that peptide-NB treatment does not affect proportions of TR1 cells amongst insulin-reactive CD4^+^ T cells in islets, spleen, or pLNs (Figure S11).

Overall, these results demonstrate that peptide-NB treatment has the potential to expand regulatory T cells, a key anti-inflammatory cell type, in the islets of NOD mice, the site of peptide-NB accumulation.

### Peptide-NBs can be developed with hybrid insulin peptides and delay diabetes onset in an antigen-specific mouse model of autoimmune diabetes

**Figure 5:**
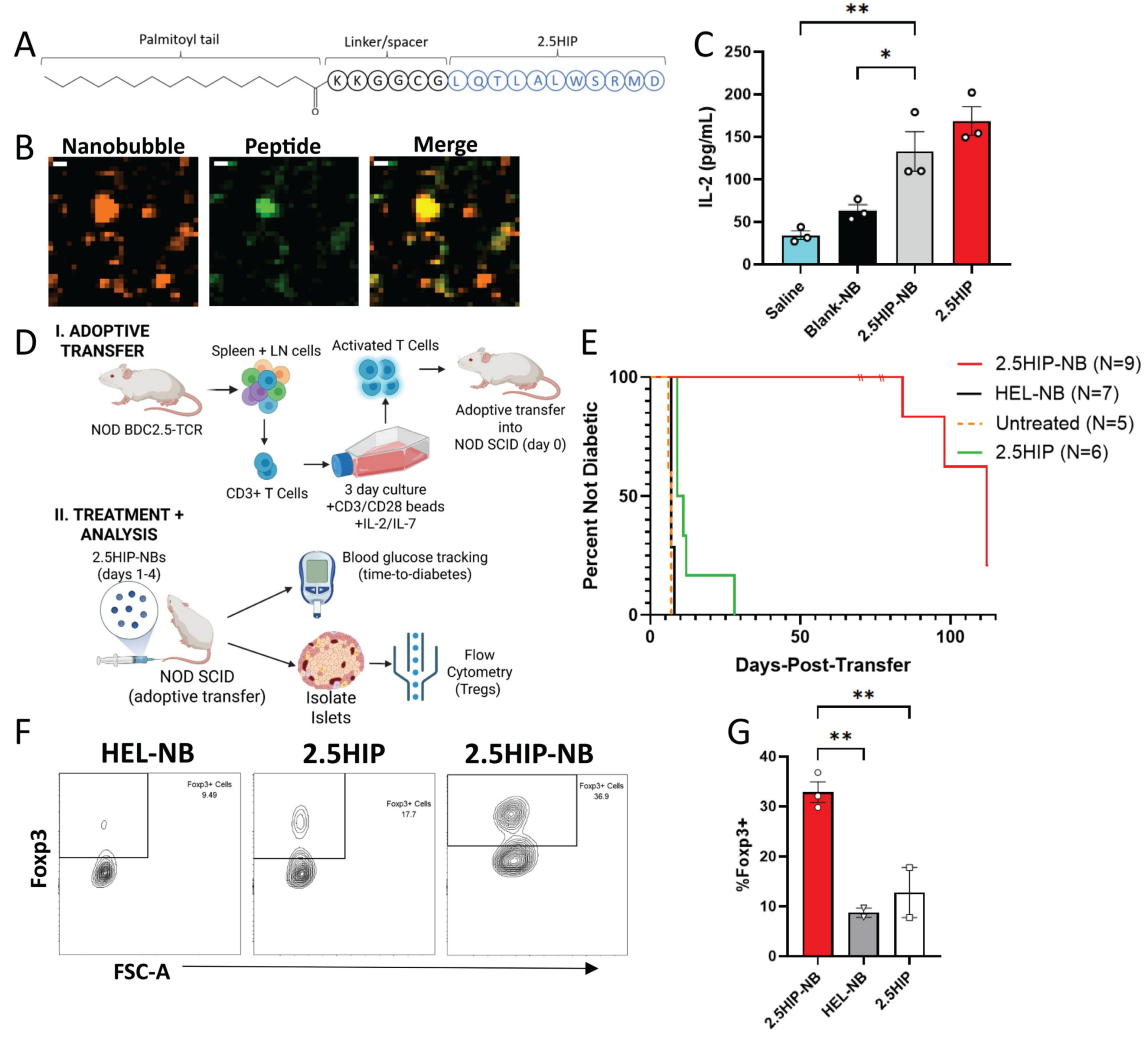
** Peptide-NBs developed with insulin-chromogranin A hybrid peptide ‘2.5HIP’ show peptide incorporation and function. A** Schematic illustrating palmitoylated HIP design. **B** Representative confocal images of rhodamine-nanobubbles (orange) containing FITC-labeled palmitoylated HIP (green). **C** Effect of nanobubble-inclusion on 2.5HIP ability to elicit T cell response. **D** Schematic illustrating experimental plan for adoptive transfer of BDC2.5 T cells and subsequent treatment and analysis. **E** Kaplan Meier curve indicating percentage of mice remaining non-diabetic for NOD-SCID mice treated 4 times with 2.5HIP-NBs, 2.5HIP, HEL-NBs (control peptide), or no treatment following adoptive transfer of activated BDC2.5-TCR CD3^+^ T Cells. **F** Representative flow cytometry gating for the identification of Foxp3^+^CD25^+^ regulatory T cells amongst islet-resident CD4^+^ T cells. **G** Proportion of Foxp3^+^ cells amongst islet-resident CD4^+^ T cells in NOD-SCID mice that had received treatment following adoptive transfer of activated BDC2.5-TCR CD3^+^ T cells. Error bars in (**C, G**) represent s.e.m. Data in (**C**) represents n = 3 lipid solutions. Data in (**E**) represent n = 9 2.5HIP-NB treated mice, n = 7 HEL-NB treated mice, n = 5 untreated mice, and n = 6 2.5HIP treated mice. n = 3 2.5HIP-NB treated mice were removed from the survival analysis at 70, 70, and 77 days-post-transfer for flow analysis (dashes). Data in (**G**) represent n = 3 2.5HIP-NB treated mice and 2 HEL-NB and 2.5HIP treated samples, where a sample contains cells pooled from 2-3 mice. Scale bars in (**B**) represent 500 nm. *p < 0.05, **p < 0.01. (**C**) p = 0.0016 comparing Saline and 2.5HIP-NB and p = 0.011 comparing Blank-NB and 2.5-HIP-NB (ANOVA). (**E**) p < 0.0001, = 0.0002, and < 0.0001 comparing 2.5HIP-NB and HEL-NB, untreated, and 2.5HIP, respectively (Log-rank test). p = 0.0004 and 0.0015 comparing 2.5HIP and HEL-NB and 2.5HIP and untreated, respectively (Log-rank test). (**G**) p = 0.0039 and 0.0075 comparing 2.5HIP-NB and HEL-NB and 2.5HIP-NB and 2.5HIP, respectively (ANOVA).

Since our strategy for incorporating insulin peptides into nanobubbles is based on lipophilic attractions rather than chemical conjugation, we tested whether this strategy holds for other peptides. Additionally, we aimed to test whether antigen-specific therapy with tolerogenic peptide-NBs can delay or prevent onset of diabetes in a monoclonal mouse model of autoimmune diabetes. Given the lack of such mouse models relevant to insulin B9-23, we focused on BDC2.5 T cells reactive to 2.5 hybrid insulin peptides (2.5HIP). We utilized the same strategy as insulin peptides – appending a palmitoyl tail with a cationic linker region – to form peptide-NBs with a 2.5HIP. Hybrid insulin peptides have been implicated as islet autoantigens and treating mice with nanoparticles containing the 2.5HIP has been shown to delay onset of diabetes using adoptive transfer of TCR-transgenic BDC2.5 T cells 20. We generated two palmitoylated peptides: “HIP1” includes a conventional version of 2.5HIP, where both the N terminus and C terminus are flanked with GGR residues. “HIP2” includes the same N terminal linker used with the insulin peptide (KKGGCG), which is longer and includes more cationic residues (Figure 5A, Figure S12A). NBs were labeled with rhodamine and peptide was labeled with FITC and peptide-NBs were imaged for colocalization to assess if efficiency of incorporation is comparable to insulin peptide. We found little to no peptide present with HIP1; however, colocalization with HIP2 was comparable to insulin peptide (Figure 5B, Figure S12B-C). Thus peptide-NBs containing 2.5HIP can be generated with the peptide linker region being necessary in addition to the palmitoyl tail. Therefore, HIP2 was used for 2.5HIP-NBs in all experiments.

We assessed if the bioactivity of HIP2 was retained following incorporation in NBs. First, we treated primary APCs with 2.5HIP-NBs, co-cultured with CD4^+^ T cells from a NOD-BDC2.5TCR mouse overnight, and measured IL-2 resulting from 2.5HIP-reactive T cell stimulation. We found that IL-2 concentration with 2.5HIP-NB treatment was similar to peptide alone and significantly greater than saline and NB controls. (Figure 5C). To further evaluate peptide function, we treated NOD mice with 2.5HIP-NBs and characterized 2.5HIP-reactive T cells from the spleen 1 w post-injection using flow cytometry and a validated 2.5HIP MHC tetramer (Figure S13). We found that treatment with 2.5HIP-NBs resulted in lower expression of CD62L amongst CD44^+^ cells, indicating peptide function in 2.5HIP-NBs, as antigen-experienced 2.5HIP-reactive T cells were adopting an effector-like rather than memory-like expression pattern as a result of 2.5HIP-NB treatment (Figure S12D-E). Finally, we determined that, following an adoptive transfer of *in vitro* activated T cells from a NOD-BDC2.5TCR mouse into NOD-SCID mice, treatment with 2.5HIP-NBs substantially delayed onset of diabetes in recipient mice (Figure 5D-E, Figure S14). We analyzed islet-infiltrating CD4^+^ T cells in these mice and found that the proportion of these T cells that were Foxp3^+^ regulatory T cells was highest in mice that had been treated with 2.5HIP-NBs (Figure 5F-G). A similar effect was observed in the pancreatic lymph nodes and spleen (Figure S12I-J). These results further indicate the effectiveness of NBs as peptide delivery vehicles for the induction of immune tolerance and delaying or preventing diabetes development in mouse models of T1D.

### The effect of insulin peptide treatment can be visualized by ultrasound measurements of microvascular permeability

**Figure 6:**
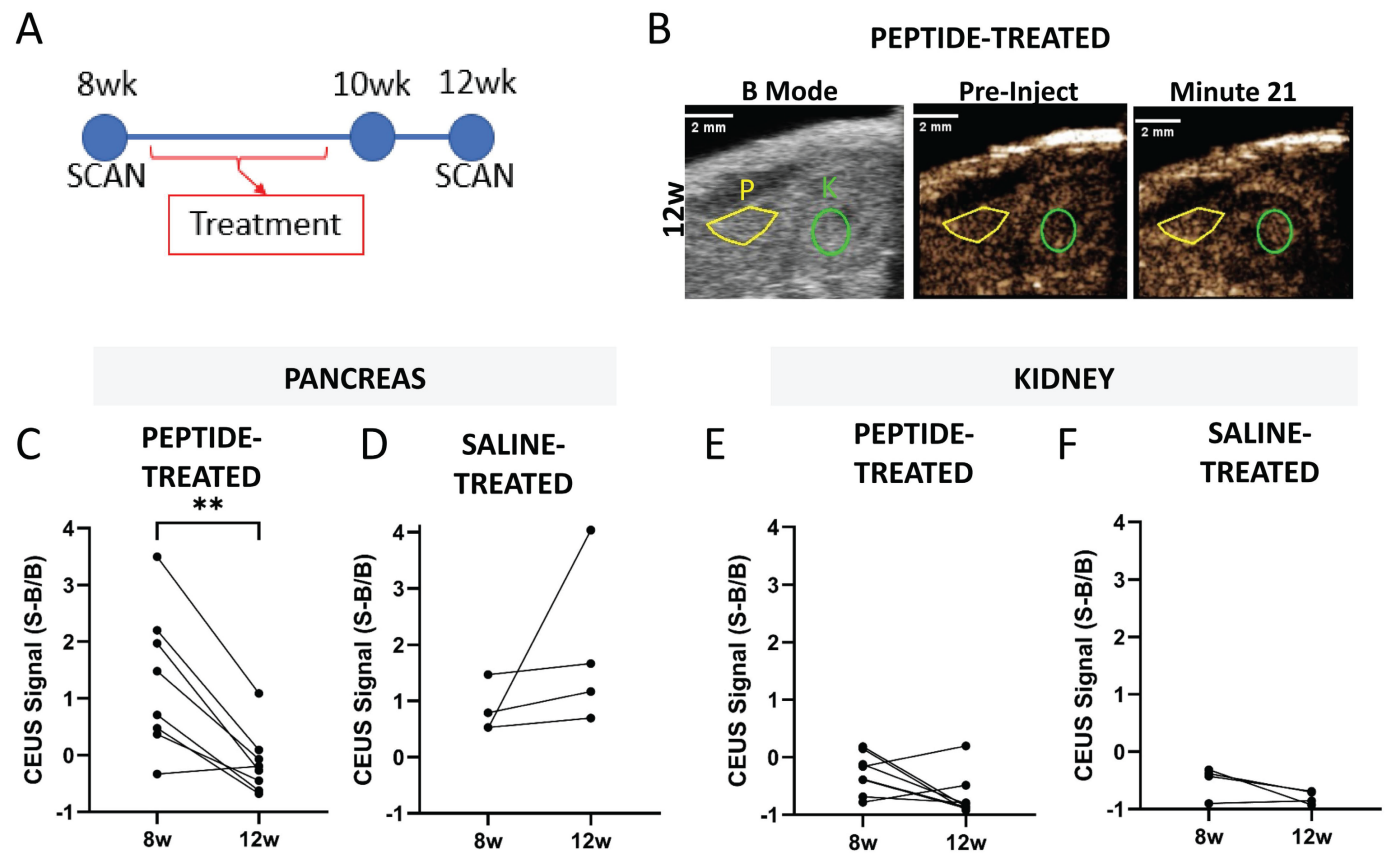
** Pancreatic NB accumulation measured with ultrasound contrast reports effects of peptide treatment in the NOD mouse pancreas. A** Schematic illustrating experimental plan. **B** Representative 12 w (post-treatment) ultrasound images including B-mode to illustrate localization of pancreas (yellow) and kidney (green) and contrast mode prior to injection of nanobubbles and 21 min post-injection. **C** Pancreatic ultrasound contrast signal averaged 20-25 min post-injection of nanobubbles prior to (8 w) and two weeks following (12 w) insulin peptide therapy in NOD mice. **D** As in (**C**), for mice that received saline rather than insulin peptide therapy. **E-F** As in (**C-D**), respectively, for the kidney. Data in (**C, E**) represent n = 8 NOD mice. Data in (**D, F**) represent n = 4 NOD mice. Scale bars in (**B**) represent 2 mm. **p < 0.01. (**C**) p = 0.002, comparing groups indicated (paired t-test, two-tailed).

Ultrasound contrast measurements of NB accumulation in the pancreas can detect changes associated with diabetes progression 39. We tested if the effects of insulin-peptide treatment on disease progression could be measured by imaging NB accumulation in the pancreas in NOD mice. NOD mice were treated five times over 2 w with palmitoylated insulin peptide and pre- and post-treatment CEUS scans were used to determine if a therapeutic effect could be measured noninvasively with ultrasound (Figure 6A). Again, the B-mode scan was used to localize pancreas and kidney (Figure 6B). Following peptide treatment, the contrast signal within the pancreas from NB accumulation significantly decreased in NOD mice (Figure 6C). There was no significant change in saline-treated mice (Figure 6D). Additionally, no change in signal was observed in the kidney under both peptide and saline treatment (Figure 6E-F).

We then tested whether insulin peptide-nanobubble treatment could impact diabetes development in the polyclonoal NOD mouse model. 6 w old female NOD mice were treated with dexamethasone together with either insulin peptide-nanobubbles, control nanobubbles, or peptide alone daily for five consecutive days (Figure S15A). Again, we found an increase in proportion of Tregs in islets with insulin peptide-nanobubble treatment (Figure S15B) and a slight increase in proportion of Tregs in pancreatic lymph nodes with insulin peptide treatment (Figure S15C). Diabetes onset was slightly delayed in a subset of NOD mice that had received insulin peptide-nanobubble (Figure S15D). Averages and standard deviations of blood glucose levels in these mice at 12-14 w (the earliest ages at which NOD mice may become diabetic) and at the 3 w prior to diabetes onset did not differ between treatment groups (Figure S16).

These results indicate that peptide-NB treatment has disease-modifying effects in NOD mice, and that the nanobubble ultrasound contrast agents have a novel potential in the detection of therapeutic-induced reversal of microvascular permeability that is associated with disease-modifying effects.

## Discussion

Most treatments being applied towards individuals with autoimmune diseases broadly affect the immune system, rather than solely diminishing the activity of autoreactive immune cells in an antigen-specific manner. This leaves patients at risk for infection and other undesirable side effects from immunosuppressive therapeutics. Because of this, antigen-specific therapies have been explored in preclinical studies for numerous conditions, including multiple sclerosis, type 1 diabetes, and rheumatoid arthritis 45. However, these therapies have largely not been translated for widespread and effective clinical use. For example*,* robustly preventing the onset of type 1 diabetes has been a persisting challenge. A promising approach that has been explored in pre-clinical models of T1D is tolerogenic antigen-specific immunotherapy, where known autoantigens are administered as exogeneous peptides in order to induce tolerance towards these antigens for prevention of diabetes onset. For instance, insulin B chain peptide mimotopes are effective at expanding insulin-reactive anti-inflammatory regulatory T cells and preventing diabetes in non-obese-diabetic mice 16, 41, 42.

**Figure 7:**
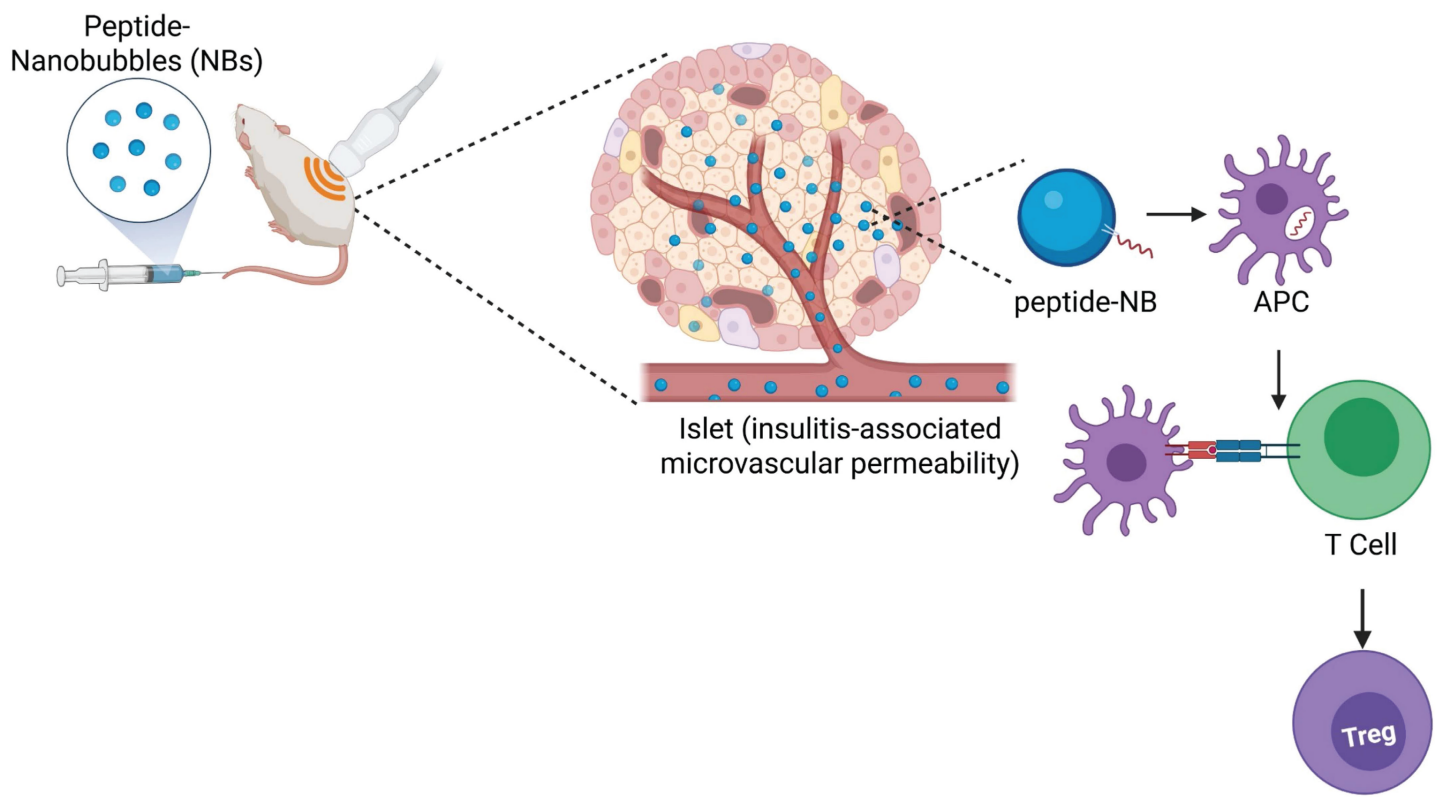
** Graphical abstract representing the overall findings from this study**.

However, results have been mixed amongst different groups and rely on a continuous infusion via surgically-implanted osmotic pump 46, 47. Approaches in humans, such as oral and nasal delivery of insulin, have not been successful to date 18, 19. Other tolerogenic peptides for T1D, such as chromogranin A peptides, hybrid insulin peptides, and co-administration of multiple peptides, have been shown to be more effective when administered with nanoparticle delivery platforms in mouse models 20-25. Therefore, we developed and utilized a novel nanobubble delivery approach for therapeutic peptide administration, which, unlike conventional nanoparticle delivery platforms, has potential as a ‘theranostic’ approach. Theranostic approaches combine therapeutic and diagnostic capabilities within the same agent, allowing for simultaneous therapy and image-based monitoring and guidance of the therapy 48, 49. Here, our goal was to test if sub-micron sized lipid-shelled nanobubble (NB) ultrasound contrast agents are an effective means of delivering peptide therapeutics, and if nanobubbles containing autoantigen peptides can modulate islet autoimmunity and prevent diabetes in mice. We demonstrated that insulin peptide mimotopes can be modified for stable incorporation in the NB lipid shell without affecting NB or peptide functions, nanobubbles can be used for peptide delivery specifically to islets, and peptide-nanobubble administration can modulate islet autoimmunity in NOD mice and prevent diabetes (Figure 7). These results demonstrate that delivering peptide autoantigens to the site of immune infiltration can be an effective means of modulating islet autoimmunity. Further, they enable development of image-guided therapeutics for T1D, allowing for image-based destruction of nanobubbles for therapeutic release, known as on demand ultrasound-mediated cavitation, visualization of therapeutic distribution, and combination with imaging-based diagnostics.

### Insulin peptide palmitoylation is an effective strategy for incorporation in nanobubbles

Our results provide a framework for incorporating peptides into lipid shelled ultrasound contrast agents. In the specific case of nanobubbles, thus far this has been limited to the delivery of small molecules 26-30. Lipid nanobubbles are similar to conventional liposomes, showing similar diameters and both including a phospholipid membrane. However, the lipid shell of nanobubbles encases an inert nonpolar heavy gas in contrast to the aqueous solution of liposomes. Additionally, liposome membranes typically include cholesterol while lipid nanobubble shell do not. As a result, nanobubbles have a less rigid membrane and are highly compressible, allowing them to dynamically change size in response to an ultrasound pressure wave, giving them utility as an ultrasound contrast agent. However, nanobubbles are less stable over time, necessitating their formulation from a precursor solution before use.

We physically incorporated palmitoylated insulin peptides in nanobubbles, with the palmitoyl tail enhancing solubility in the NB lipid shell, for stable incorporation without affecting peptide properties. The palmitoylated peptide is incorporated with lipid prior to hydration and formation of nanobubbles. It is stabilized in the lipid membrane likely by a combination of lipophilic and ionic interactions. Stability of incorporation can also be attributed to the six-residue linker region (Figure S12); while the palmitoyl tail anchors the peptide into the fatty acid “tail” regions of the phospholipid shell, the net-cationic linker residues (lysine) could be contributing as well by anchoring the peptide into the net-anionic (phosphatidic acid) “head” regions of the phospholipid shell. This approach, as opposed to incorporation of non-palmitoylated peptides or covalent linkage of peptides, optimally balances stability of incorporation with retention of peptide bioactivity and antigen presentation. We find that peptide palmitoylation increases colocalization with NBs without significantly altering bioactivity (Figure 1) and allows for peptide to be delivered to NOD islets by NBs (Figure 2). While covalent attachment may result in highly stable peptide incorporation, the ability for the peptide to be processed and presented by antigen-presenting cells and stimulate insulin-reactive T cell receptors is almost completely abrogated, even if NBs are ablated (Figure S4). We used an *in vitro* IL-2 secretion assay, saturating splenic antigen-presenting cells with peptide, to determine if palmitoylated peptide could still be processed and presented by antigen presenting cells. This was not intended to serve as an *in vitro* tolerance induction experiment or to study peptide processing and presentation by APCs in detail.

Our primary objective is for autoantigen peptides to be sufficiently incorporated in nanobubbles to allow for islet-targeted peptide delivery. Our results show that NBs can target peptide delivery to islets in NOD mice (Figure 2, Figure S6). This builds upon prior work from our group that demonstrates increased microvascular permeability near islets of these mice, allowing for accumulation of NBs in islets 39, 50. While this “enhanced-permeability and retention” phenomenon has been described extensively in the context of tumor vessels, it has not been as thoroughly explored in the context of inflammation and insulitis 51. We show this accumulation of NBs by histological measurement of pancreatic NB accumulation and utilize this accumulation as a means of targeting peptide delivery to islets.

### Peptide-nanobubble distribution can be validated in real-time with ultrasound contrast

While other nano-scale constructs could be utilized for peptide delivery to islets, nanobubble ultrasound contrast agents carry several unique benefits. They can be visualized in real-time, noninvasively, using ultrasound and are capable of on demand ultrasound-mediated cavitation, meaning they can be burst noninvasively using ultrasound to aid in release of cargo 52-59. Much of nanobubble function as ultrasound contrast agents relies on the properties of their shell – since we are adding a novel element into this lipid shell, it is crucial to verify that the inherent characteristics and functionality of the NBs are not affected by peptide 31. Our results show that peptide does not substantially affect NB physical properties, ability-to-burst, or echogenicity *in vitro* (Figure 1, Figure 3, Figure S2, Figure S3).

The echogenic properties and applications of NBs are also unaffected by peptide *in vivo*. These results build upon our prior work that measured pancreatic accumulation of NBs in NOD mice with ultrasound contrast, which provides a novel diagnostic for presymptomatic T1D 39. Ultrasound-based measurement of pancreatic NB accumulation with both peptide-NBs and plain NBs showed similar levels of NB accumulation and ultrasound contrast in the pancreas, indicating that peptide does not inhibit NB echogenicity *in vivo* or pancreatic NB accumulation (Figure 3). These results further validate histological data demonstrating accumulation of peptide-NBs. The accumulated signal is ~11% of the peak signal, indicating that while the majority of nanobubbles are rapidly cleared and/or depleted, enough peptide-nanobubbles accumulate in the pancreas for identification with ultrasound contrast and tolerance induction.

In this study, we conduct ultrasound scans in NOD mice at age 9 w (Figure 3), while treating NOD mice at age 9 w (Figure 4) or 6 w (Figure S15) – see next subsection. These time points are prior to diabetes (‘presymptomatic’), yet at old enough for insulitis to have started. While we did not conduct ultrasound scans with peptide-nanobubbles at earlier ages, when insulitis may be initiated, we have previously observed higher ultrasound contrast from accumulated unloaded nanobubbles in the NOD pancreas compared to the pancreas of control mice at 4w 39. Further, the pancreas is in close proximity to the intestine which has a high background signal and can move within the field of view. All scans were preceded by background scans to quantify and normalize to this background signal. Prior work, via the delivery of saline alone, established that such a background does not influence ultrasound measurements of nanobubble accumulation 39. Importantly islet immune infiltration (insulitis) and beta-cell mass decline is heterogeneous in T1D. We previously assessed the impact of insulitis and residual beta-cell mass on nanobubble accumulation and did not observe any significant association for these factors in islet-to-islet variation in nanobubble accumulation 39.

Another key potential benefit of ultrasound measurement of pancreatic NB accumulation is the ability to measure therapeutic-induced reversal of vascular permeability. Notably, the presence of circulating autoantibodies, the gold-standard of presymptomatic diagnosis of T1D, does not necessarily change upon induction of immune tolerance and prevention of diabetes onset 60. Our results show that ultrasound contrast from accumulated NBs in the pancreas decreases in response to therapeutic intervention with insulin peptide mimotopes (Figure 6). These results indicate that contrast enhanced ultrasound is both sensitive enough to predict diabetes onset in NOD mice, and also sensitive enough to elucidate therapeutic-induced changes in disease progression. Nanobubbles are therefore useful tools for both the delivery of insulin peptides and assessment of the effect of peptide delivery on islet immunity.

### Peptide-nanobubble treatment modulates islet autoimmunity in pre-clinical models

Our data showed that delivery of peptide with peptide-NBs in NOD mice at 9 w (when islet immune infiltration is expected but diabetes onset is not) has anti-inflammatory effects in *islets* (Figure 4) and has disease-modifying effects in an adoptive transfer model of autoimmune diabetes (Figure 5). This indicates that the peptide is bioactive *in vivo*, peptide delivery to islets allows for islet-specific immune modulation, and peptide-NBs impact regulatory T cell balance in islets. Cells were analyzed 3 w after treatment, providing confidence that this immunomodulatory effect is not just transient, and peptide-NBs can have lasting anti-inflammatory effects on the immune composition of the islet. It will be beneficial in the future to examine changes in islet immune composition over time following treatment to better understand the longevity of these beneficial immune responses, as most female NOD mice are expected to develop diabetes later than the age at which cells were analyzed. We also anticipate that treating at a later age would not be effective, as a substantial amount of beta-cell destruction will have already occurred. Therefore, we treated NOD mice at ages where insulitis is expected to have started but severe hyperglycemia is not, as other groups utilizing similar peptides have done 41. At 9 w old – when NOD-Foxp3GFP mice were treated to assess islet-resident regulatory T cells (Figure 4), mice are still expected to have some dysglycemia despite being non-diabetic 61, 62.

Interestingly, while peptide-NB treatment had an anti-inflammatory effect in islets, it had no effect in nearby pancreatic lymph nodes (pLNs). On the other hand, treatment with palmitoylated peptide alone without NBs did have such an effect in pLNs, with the proportion of Tregs amongst insulin-reactive CD4^+^ T cells being highest for peptide-treated mice. While these opposing results are surprising given the strong influence exerted by pLNs on islet immunity, they may highlight the beneficial effects of targeted peptide delivery to the site of immune infiltration compared to systemic peptide delivery. We also demonstrated that non-insulin-reactive Tregs are not affected by insulin peptide-NB treatment, indicating that peptide-NB treatment has antigen-specific effects and may not substantially affect other T cells via a ‘bystander’ effect. In the future, it will also be beneficial to examine effects on other immune cells, such as dendritic cells, as these cells could play crucial roles in the mechanism by which peptide-nanobubble treatment results in an expansion of Tregs.

We also applied the peptide-nanobubble platform to a BDC2.5 adoptive transfer model to determine if a therapeutic effect occurs when all T cells are reactive to the antigen we are delivering. This allowed us to elucidate if the platform is effective at inducing immune tolerance at a baseline level prior to testing in a more complex model. While we did not have a monoclonal mouse model in which to test insulin B:9-23 reactivity, 2.5HIP hybrid insulin peptides are a relevant autoantigen in human T1D. Having demonstrated that peptide-NBs can also induce tolerance with other peptides (2.5HIP), it would also be beneficial to explore incorporation of multiple relevant tolerogenic peptides in the same nanobubble formulation. While diabetes onset in NOD mice requires insulin autoimmunity, T cells reactive to other islet autoantigens play roles in T1D progression in both mice and humans; most individuals who develop T1D have circulating autoantibodies against multiple islet autoantigens 60. Because of this, nanoparticle-based delivery of multiple antigens has been shown to be more effective at preventing T1D onset in mice 22. This is especially important considering the differences we observe between delay of diabetes onset in the NOD mouse model and the BDC2.5 adoptive transfer mouse model. While there are numerous differences between these two models in disease pathogenesis, a key difference is whether disease is driven by a monoclonal (BDC2.5 A.T.) or polyclonal (NOD) population of T cells. The delivery of only a single antigenic peptide could partially explain why we see much more prominent differences between treatment groups in the BDC2.5 adoptive transfer model compared to the NOD model.

Future work will also focus on the mechanism underlying the antigen-specific expansion of regulatory T cells. Our data suggest this may occur locally amongst immune cells in the peripheral tissue, given the lack of an effect in pLNs upon peptide-nanobubble treatment. This suggests that APC priming may play an important role since differences are observed when antigens are delivered with nanomaterials, which may prime APCs differently. However, further investigation is required to fully understand this mechanism.

### Potential for future applications and translation

These results serve as an important demonstration of usage of NBs for peptide delivery, local tolerance induction and delay/prevention of autoimmunity and disease. In the future, peptide-NBs should be extended to other autoimmune diseases, to test whether delivery to other sites of immune infiltration can expand other antigen-specific T regulatory cells and modulate autoimmunity. There are also other unique benefits offered by utilizing NBs as delivery vehicles in comparison to injection of soluble peptide. For instance, NBs could be used for co-delivery and targeting of multiple types of therapeutic molecules simultaneously. This could include lipophilic small molecule drugs, such as rapamycin and other mTOR inhibitors, which have a synergistic effect with tolerogenic peptide therapy in mouse models of T1D 41.

There are also unique benefits to specifically utilizing a nanobubble for therapeutic delivery. Ultrasound contrast agents are capable of on demand ultrasound-mediated cavitation as a means of releasing encapsulated therapeutics at a desired time point 52-59. Future studies should explore the impact of bursting. While this could enhance therapeutic efficacy by allowing for spatially and temporally controllable therapeutic release, our approach of not bursting nanobubbles could be more effective in the case of peptide antigens, as other groups have shown that encapsulation of tolerogenic peptides in nanocarriers with an anionic surface charge is beneficial in the context of peptide uptake and presentation 20, 63. Additionally, use of nanobubbles allows for the dosage to be dynamically tuned to the individual, as nanobubble biodistribution within the region underneath the ultrasound transducer can be visualized in real-time, allowing for increases in dosage depending on the extent of pancreatic distribution. A*ctive targeting* to disease sites achieved by a targeting ligand appended to the nanobubble is also feasible. This has been done for targeting of nanobubbles to OVCAR-3 tumors in mice as well as targeting of iron oxide nanoparticles to pancreatic beta-cells in mice 64, 65. One limitation of nanobubbles is encapsulation of drugs exclusively in the lipid shell, restricting the payload of cargo that can be carried by the nanobubble. However, the relatively high surface area to volume of NBs, optimizes this compared to conventional microbubbles. Finally, peptides could also be incorporated in phase-change nanodroplet ultrasound contrast agents, which are also submicron and can be vaporized into microbubbles noninvasively using ultrasound. Prior studies have indicated that these contrast agents can also accumulate in the NOD pancreas and this accumulation can be quantified using ultrasound with a substantially lower contrast agent dosage 39, 50.

This design also carries potential for future human translation. Microbubble ultrasound contrast agent formulations similar to our formulation, such as DEFINITY which includes a perfluorocarbon gas core, lipid shell, and contains a submicron portion, have been FDA approved and are generally safe, cost-effective, and easy to use. Microbubbles are also currently being applied to imaging pancreatic vascular dynamics in humans at risk for T1D (ClinicalTrials.gov #NCT05482321). While these clinical contrast agents are more polydisperse than NBs, we have previously demonstrated selective accumulation of phase-change ultrasound contrast agents generated from polydisperse DEFINITY bubbles in the pancreas 50. Phase-change contrast agents are also currently being applied to cancer therapy in humans (ClinicalTrials.gov #NCT04021277). While nanobubble accumulation in the pancreas has not yet been evaluated in humans, other groups have shown that iron oxide nanoparticle-based MRI contrast agents accumulate in the pancreas of humans developing T1D, indicating that this approach may be relevant for human T1D 36, 37. A lower center frequency is typically used when imaging humans due to the higher imaging depth required; while the small animal ultrasound imaging parameters used in this study include a higher frequency, the human pancreas can readily be located and nanobubbles can be imaged at clinically relevant frequencies. Finally, while it is extremely important to develop preventative therapies for T1D that address the underlying autoimmunity that drives T1D, artificial islet approaches are also promising and carry the potential to treat T1D *after* diagnosis, especially when combined with immunomodulatory approaches. Indeed, nanoparticle-mediated tolerogenic therapy can be used to help prevent rejection of artificial and transplanted islets and can improve their function 66, 67.

## Conclusions

In summary, we present a novel theranostic approach for delivering therapeutic peptides to islets in non-obese-diabetic mice. Islet microvascular permeability is increased during disease progression in these mice, allowing for accumulation of peptide-nanobubbles in NOD islets. Almost all functionality of both the peptide and nanobubble are retained following formation of peptide-NBs and treatment of NOD mice with peptide-NBs expanding anti-inflammatory insulin-reactive regulatory T cells in islets. This represents a novel and innovative approach for enhancing the efficacy of peptide autoantigens for tolerance induction and could be extended to other autoimmune diseases and feasibly translated to human type 1 diabetes.

## Supplementary Material

Supplementary Figures S1-16, including in-depth characterization of nanobubble physical properties and dynamics of peptide encapsulation and release, analyses of non-palmitoylated and chemically conjugated peptides, full tissue section images, nanobubble circulation dynamics and clearance, flow cytometry gating and additional analyses, validation of peptide-MHC tetramers, and in-depth analysis of blood glucose time-courses in mice.

## Data Availability

All Imaging data (microscopy, ultrasound) will be available at the EMBL-supported BioImage Archive following publication. All data included in this article are available upon request.

## Abbreviations

T1D: type 1 diabetes
NB: nanobubble
NOD: non-obese diabetic
HIP: hybrid insulin peptide
CEUS: contrast-enhanced ultrasound
Pal/Palm: palmitoyl
PBS: phosphate-buffered saline
RMM: resonant mass measurement
CHI: contrast harmonic imaging
MI: mechanical index
H&E: Hematoxylin and Eosin
HEL: hen egg lysozyme
EE: encapsulation efficiency
TCR: T cell receptor
APC: antigen-presenting cell
Treg: regulatory T cell
pLN: pancreatic lymph node
TR1: T regulatory type 1 cell
A.T.: adoptive transfer
I.P.: intraperitoneal
IV: intravenous
GFP: green fluorescent protein
MRI: magnetic resonance imaging
MHC: major histocompatibility complex
pMHC: peptide-MHC complex
PEG: (poly) ethylene glycol
SCID: severe combined immunodeficiency
FSC: forward scatter
SSC: side scatter
MHz: megahertz
