# Supplementary material for: Submicron-sized ultrasound contrast agents as therapeutic peptide delivery vehicles in type 1 diabetes
Source: Theranostics. 2026 May 11;16(12):6692–712. doi: 10.7150/thno.126925 (PMC13231989; doi:10.7150/thno.126925)
Supplement: Supplementary file 1 — Supplementary Figures S1-16, including in-depth characterization of nanobubble physical properties and dynamics of peptide encapsulation and release, analyses of non-palmitoylated and chemically conjugated peptides, full tissue section images, nanobubble circulation dynamics and clearance, flow cytometry gating and additional analyses, validation of peptide-MHC tetramers, and in-depth analysis of blood glucose time-courses in mice. [file thnov16p6692s1.pdf]

Supplementary Information

Submicron-sized ultrasound contrast agents as therapeutic peptide delivery vehicles in type 1 diabetes.

Mark Ciccaglione, Eric Abenojar, Theresa Kosmides, James E. DiLisio, Kristen A. McDaniel, Kathryn Hartmoore, Anne Gresch, Dillon K. Jarrell, David G. Ramirez, Maura Sticco-Ivins, Aaron W. Michels, Kathryn Haskins, Agata A. Exner, and Richard K.P. Benninger

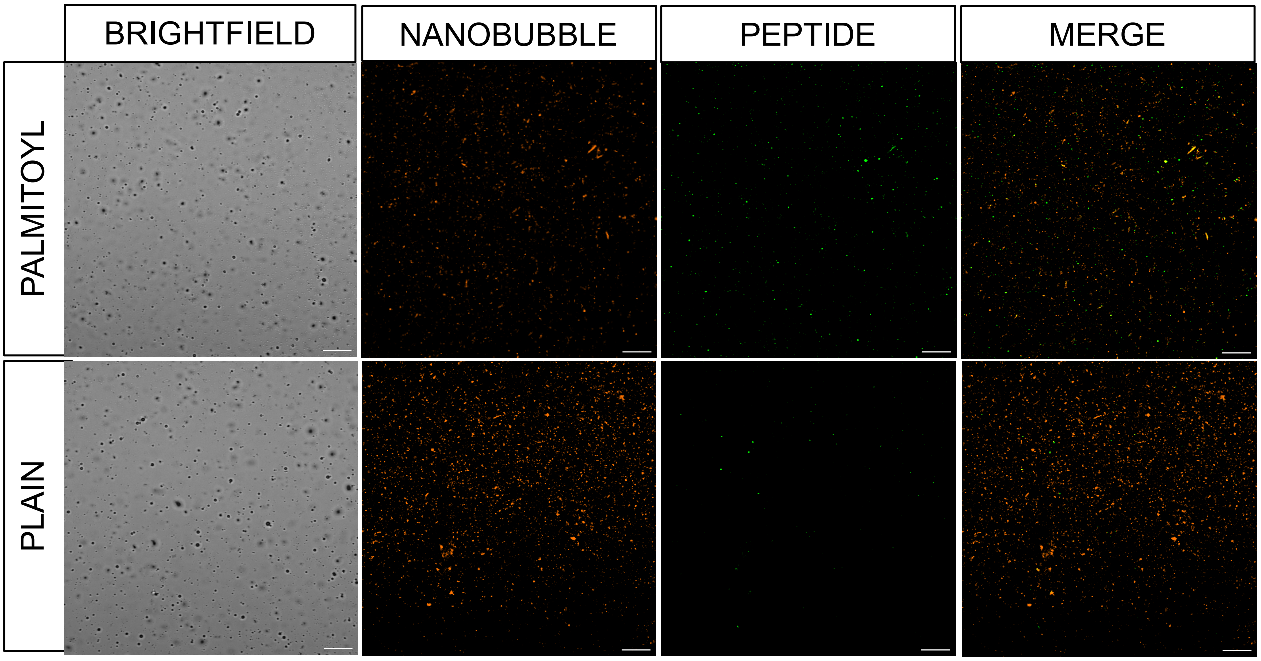

Figure S1. Effect of insulin peptide palmitoylation on incorporation in nanobubbles. Representative confocal images of rhodamine-nanobubbles (orange) containing FITC-labeled palmitoylated peptide (green; top) or non-palmitoylated “plain” peptide (green; bottom). Scale bars represent 10 μm.

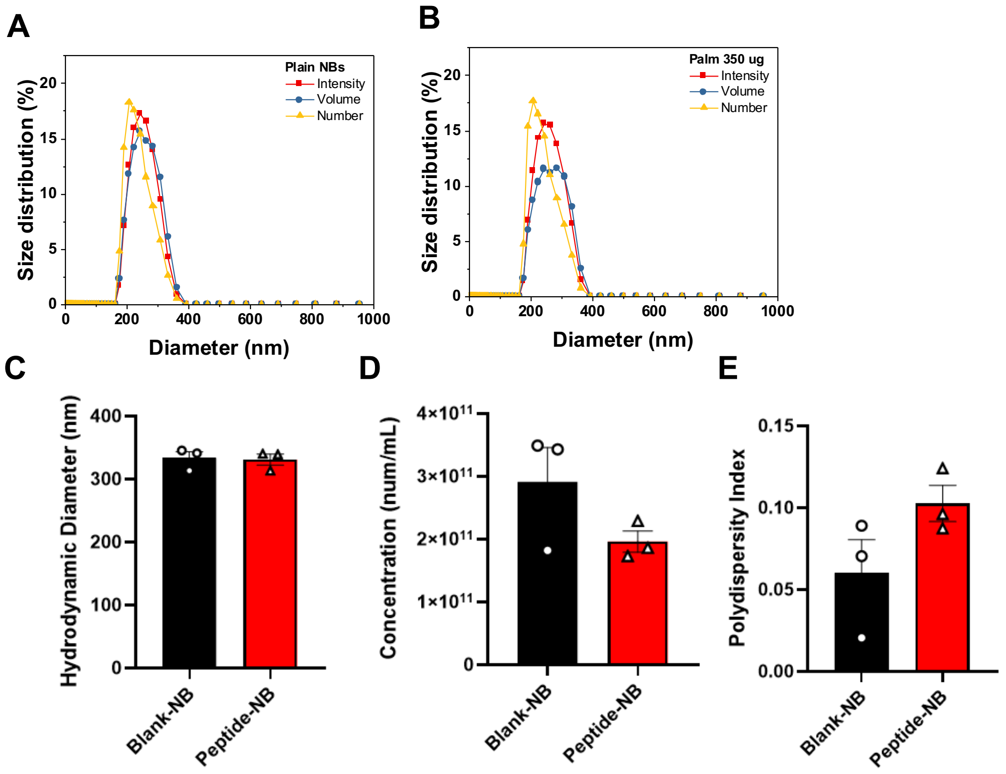

Figure S2. Effect of insulin peptide inclusion on nanobubble properties. A Nanobubble size distribution based on intensity, volume, and number with no peptide. **B** As in (**A**), with inclusion of palmitoylated insulin peptide. **C** Nanobubble average hydrodynamic diameter with and without peptide inclusion. **D** As in (**C**), for nanobubble concentration. **E** As in (**D**), for polydispersity index. Error bars in (**C-E**) represent s.e.m. Data in (**C-E**) represent n = 3 lipid solutions.

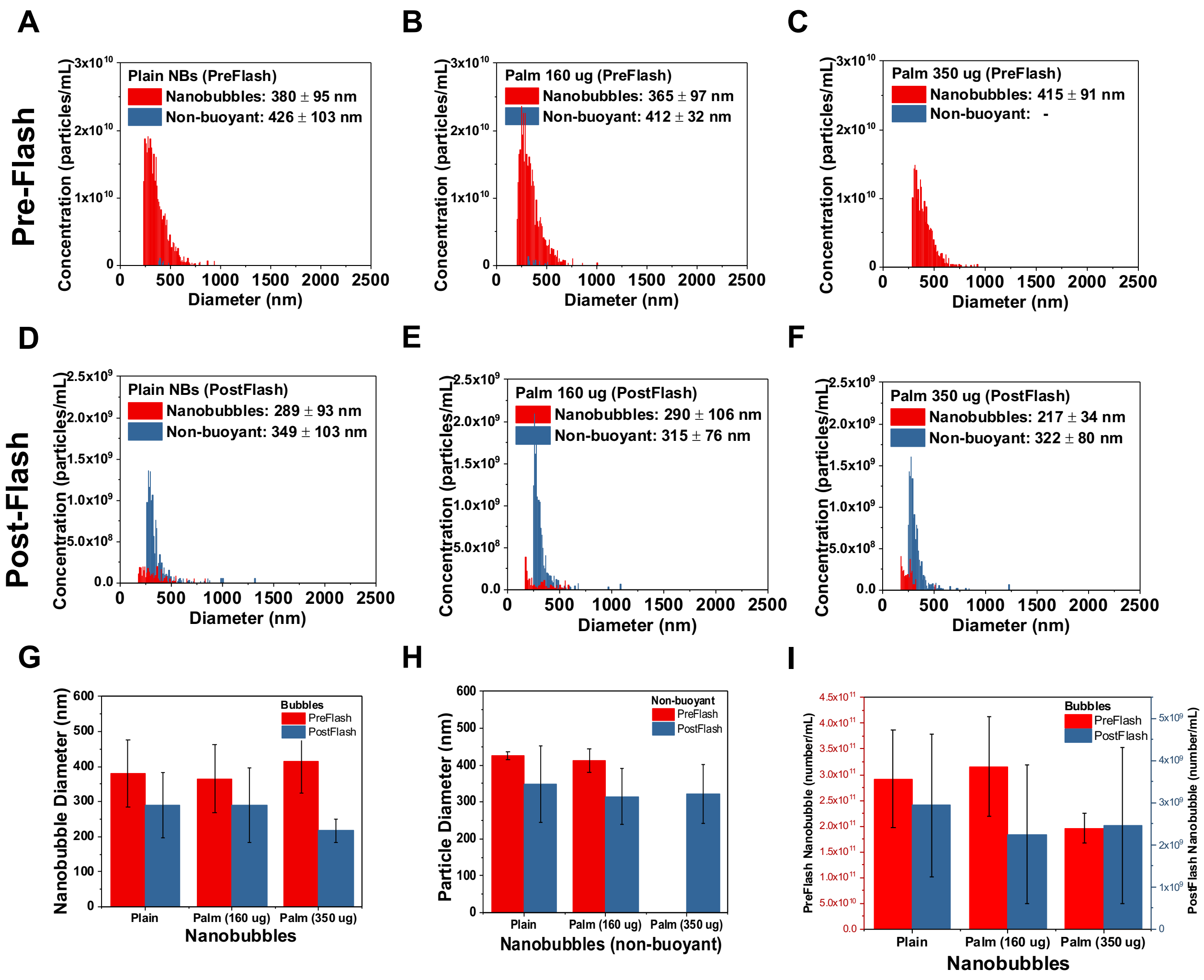

Figure S3. Effect of nanobubble peptide inclusion on formation of buoyant (bubble) vs. non-buoyant particles. A Size distribution of nanobubbles with buoyant particles (i.e. bubbles) and non-buoyant particles (i.e. lipid particles) shown separately. **B** As in (**A**), with a low concentration of palmitoylated insulin peptide. **C** As in (**A**), with a high concentration of palmitoylated insulin peptide. **D-F** As in (**A-C**), after a high mechanical-index ultrasound pulse ("flash") to burst bubbles. **G** Mean nanobubble (buoyant particle) diameter of nanobubbles containing no peptide ("plain), or a low (160 µg/mL) or high (350 µg/mL) concentration of palmitoylated insulin peptide. **H** As in (**G**), for mean non-buoyant particle diameter. **I** Mean nanobubble concentration of nanobubbles containing no peptide ("plain), or a low (160 µg/mL) or high (350 µg/mL) concentration of palmitoylated insulin peptide, both before (red bars; left y-axis) and after (blue bars; right y-axis) flash. Error bars in (**G-I**) represent s.e.m. Data represent n = 3 lipid solutions.

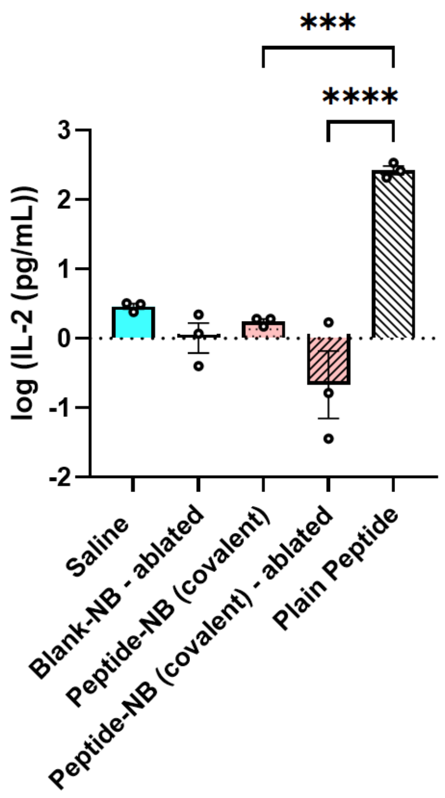

Figure S4. Covalently linkage of insulin peptides to nanobubbles eliminates peptide bioactivity. Mean log(IL-2) production from an insulin-reactive T cell hybridoma (PCR-10) stimulated with insulin peptide covalently attached to nanobubbles. Error bars represent s.e.m. Data represent n = 3 lipid solutions. ****$p < 0.0001$.

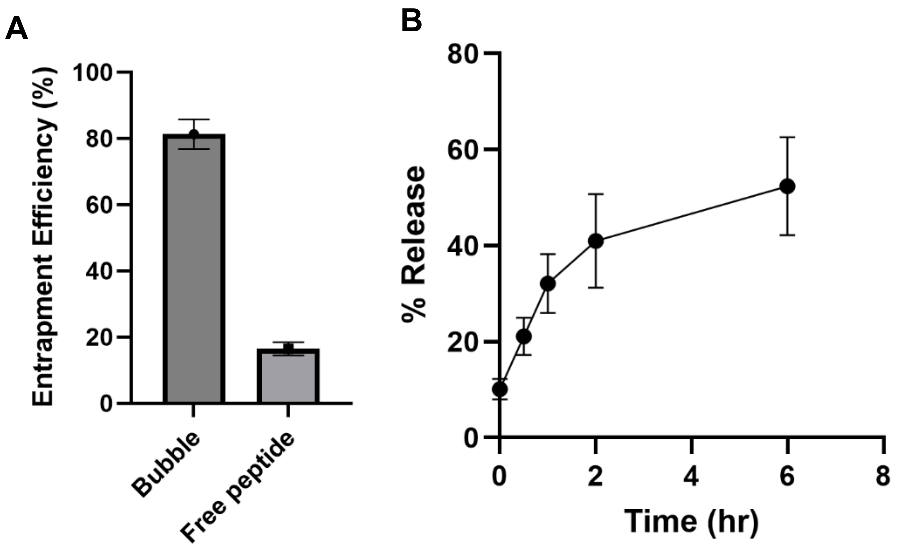

Figure S5. Efficiency of peptide incorporation and dynamics of peptide release from nanobubbles. A Percentage of initial peptide effectively entrapped in nanobubbles (‘Bubble’) and not entrapped in nanobubbles (‘Free peptide’). **B** Percentage of entrapped peptide released from nanobubbles over time. Data in (**A, B**) represent n = 3 lipid solutions.

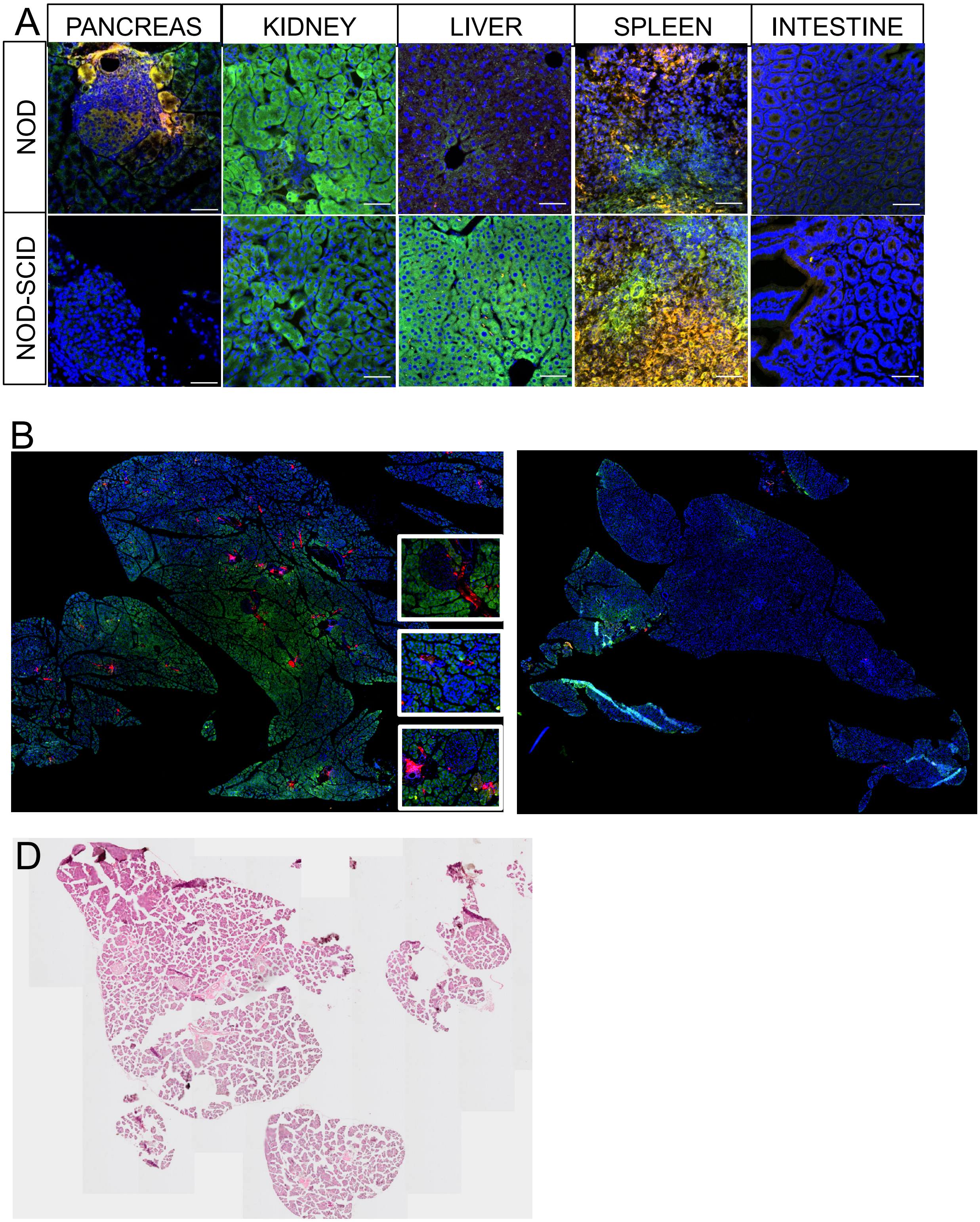

Figure S6. Insulin peptide-nanobubble biodistribution. A Representative confocal images of sections from abdominal organs a 10 w old NOD mouse 30 min post-injection of rhodamine-labeled nanobubbles (orange) containing FITC-labeled palmitoylated insulin peptide (green),

counterstained with DAPI (blue). **B** Slide scan of a 10 w old NOD mouse 30 min post-injection of rhodamine-labeled nanobubbles (orange) containing FITC-labeled palmitoylated insulin peptide (green), counterstained with DAPI (blue). **C** As in (**B**), for a B6 mouse. **D** As in (**B**), with H&E staining. Scale bars in (**A**) represent 50 µm.

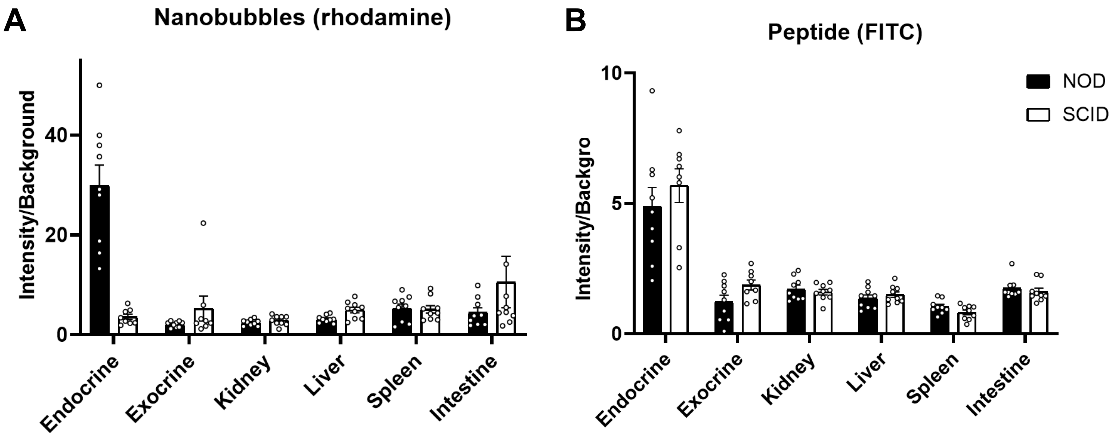

Figure S7. Fluorescent intensity of nanobubbles. Normalized fluorescent intensity of rhodamine-nanobubbles in abdominal tissues 30 min post-injection where 'endocrine' represents nanobubble-labeled regions for islets that have nanobubble accumulation. **B** As in (**A**), for FITC (palmitoylated peptide) intensity. Error bars represent s.e.m. 'Endocrine' represents nanobubble-labeled regions for islets that have nanobubble accumulation. Data represent N = 9 NOD and N = 9 SCID mice (8 SCID pancreas measurements).

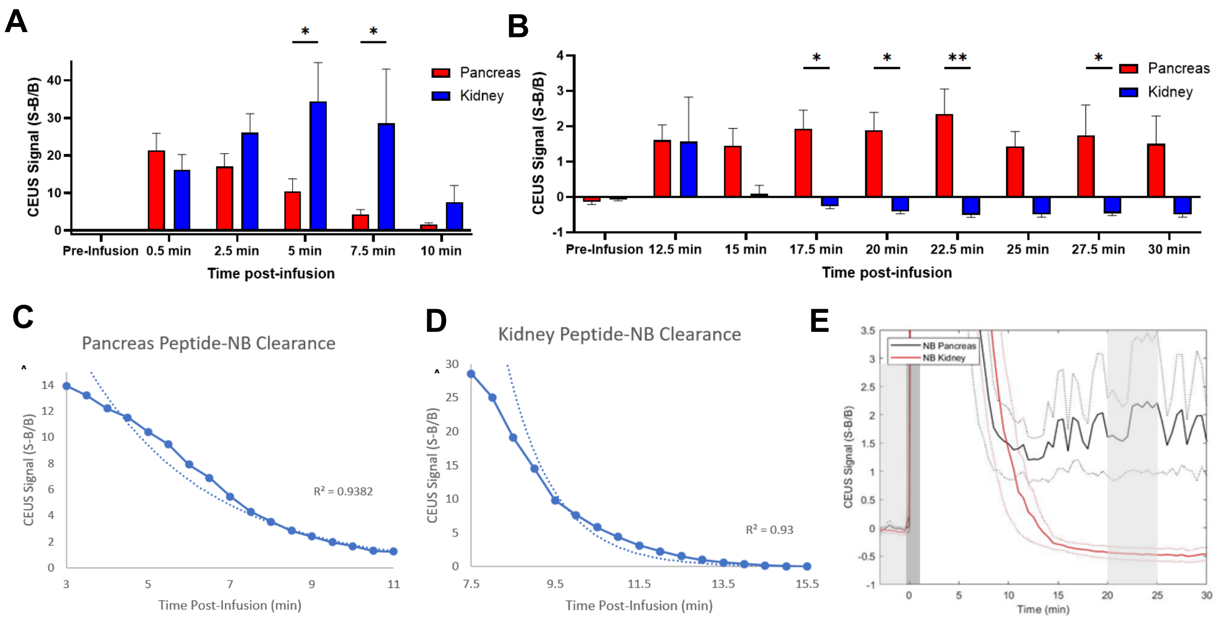

Figure S8. Nanobubble dynamics in the pancreas and kidney. A Background-normalized ultrasound contrast signal in the pancreas and kidney at varying time-points following infusion of peptide-nanobubbles in 9w NOD mice between 0.5-10 min post-infusion. **B** As in (**A**), for 12.5-30 min post-infusion. **C** Exponential decay model fit to the average ultrasound contrast signal in the pancreas of NOD mice infused with peptide-nanobubbles (half-life = 125 s). **D** As in (**C**), for kidney (half-life = 40 s). **E** Time-course of background-normalized ultrasound contrast signal in NOD mice (N = 6) following injection of nanobubbles not containing peptide. Error bars and lines represent s.e.m. Data in (**A-D**) represent N = 7 mice and data in (**E**) represent N = 6 mice. *p < 0.05, **p < 0.01. (**A**) p = 0.0330 and 0.0292 comparing pancreas and kidney at 5 and 7.5 min, respectively (ANOVA). (**B**) p = 0.0249, 0.0152, 0.0011, and 0.0233 comparing pancreas and kidney at 17.5, 20, 22.5, and 27.5 min, respectively (ANOVA).

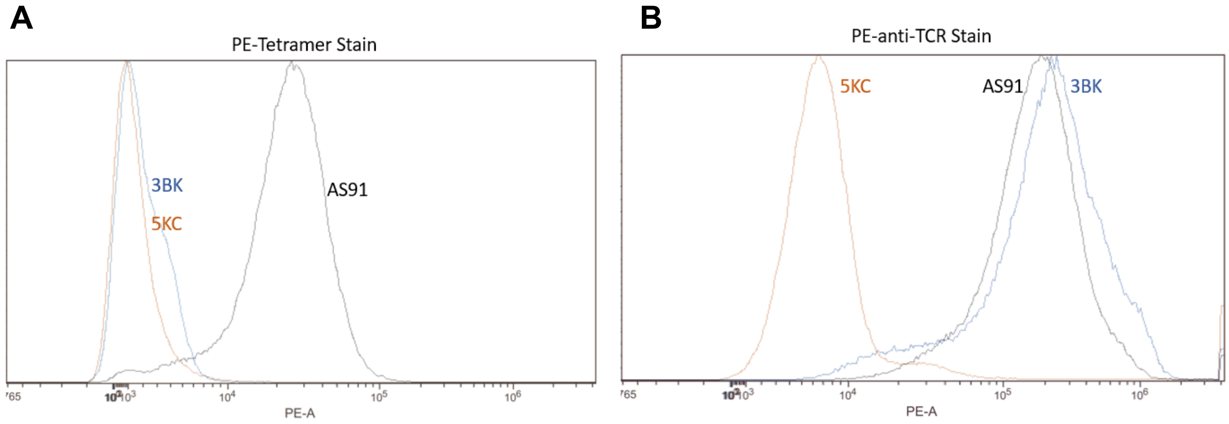

Figure S9. Validation of insulin MHC tetramer binding specificity using T cell hybridomas. **A** Histogram of signal intensity from T cell hybridomas stained with PE-labeled MHC tetramers specific to insulin-reactive T-cell receptors (TCRs), including one hybridoma with insulin-reactive TCRs (AS91), one with irrelevant TCRs (3BK) and one with no TCRs (5KC). **B** As in (**A**), with cells stained with a PE-labeled anti-TCRβ antibody.

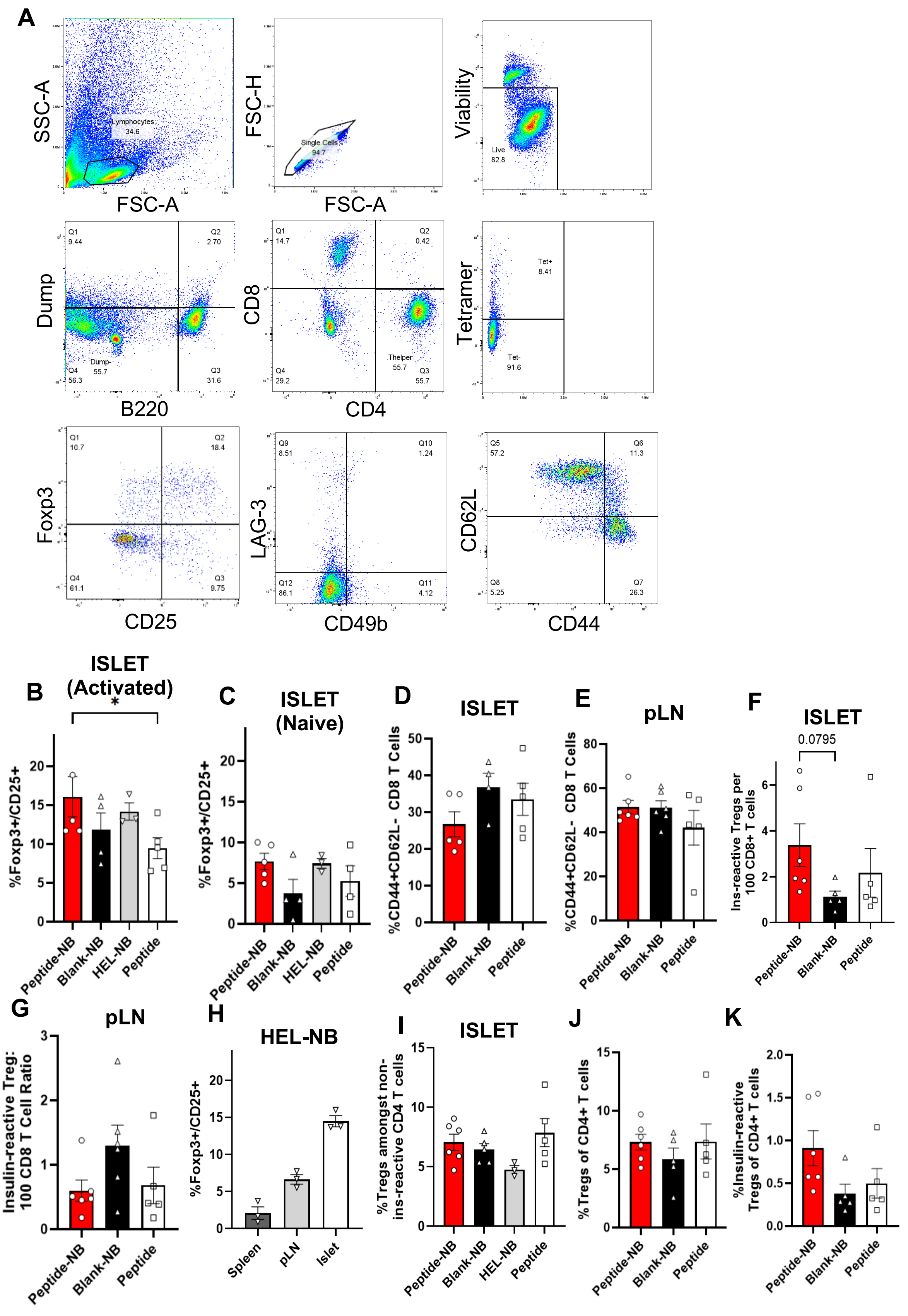
A
SSC-A
FSC-A
Lymphocytes
34.6
FSC-H
FSC-A
Single Cells
94.7
Viability
Live
82.8
Dump
B220
Q1
9.44
Q2
2.70
Q4
56.3
Dump-
55.7
Q3
31.6
CD8
CD4
Q1
14.7
Q2
0.42
Q4
29.2
Thelper
55.7
Q3
55.7
Tetramer
Tet+
8.41
Tet-
91.6
Foxp3
CD25
Q1
10.7
Q2
18.4
Q4
61.1
Q3
9.75
LAG-3
CD49b
Q9
8.51
Q10
1.24
Q12
86.1
Q11
4.12
CD62L
CD44
Q5
57.2
Q6
11.3
Q8
5.25
Q7
26.3
B
ISLET (Activated)
*
%Foxp3+/CD25+
Peptide-NB
Blank-NB
HEL-NB
Peptide
C
ISLET (Naive)
%Foxp3+/CD25+
Peptide-NB
Blank-NB
HEL-NB
Peptide
D
ISLET
%CD44+CD62L- CD8 T Cells
Peptide-NB
Blank-NB
Peptide
E
pLN
%CD44+CD62L- CD8 T Cells
Peptide-NB
Blank-NB
Peptide
F
ISLET
0.0795
Ins-reactive Tregs per 100 CD8+ T cells
Peptide-NB
Blank-NB
Peptide
G
pLN
Insulin-reactive Treg: 100 CD8 T Cell Ratio
Peptide-NB
Blank-NB
Peptide
H
HEL-NB
%Foxp3+/CD25+
Spleen
pLN
Islet
I
ISLET
%Tregs amongst non-ins-reactive CD4 T cells
Peptide-NB
Blank-NB
HEL-NB
Peptide
J
%Tregs of CD4+ T cells
Peptide-NB
Blank-NB
Peptide
K
%Insulin-reactive Tregs of CD4+ T cells
Peptide-NB
Blank-NB
Peptide

Figure S10. Additional analysis of regulatory T cells in NOD mice. A Representative flow cytometry gating. **B** Proportion of Tregs amongst insulin-reactive $CD4^+CD44^+CD62L^-$ T cells in 9 w old NOD mice that had been treated with peptide-nanobubbles, blank nanobubbles, HEL-nanobubbles (irrelevant control peptide) or peptide in islets, **C** As in (**B**), for Tregs amongst insulin-reactive $CD4^+CD44^-CD62L^+$ T cells. **D** Proportion of $CD44^+CD62L^-$ cells amongst $CD8^+$ T cells in islets. **E** As in (**D**), for pancreatic lymph nodes. **F** Ratio of insulin-reactive Tregs to 100 $CD8^+CD4^-$ T cells in islets. **G** As in (**F**), for pancreatic lymph nodes. **H** Proportion of Tregs amongst insulin-reactive $CD4^+$ T cells of NOD mice treated with HEL-nanobubbles. **I** As in (**B**), for Tregs amongst non-insulin-reactive $CD4^+$ T cells. **J** Proportion of Tregs amongst all $CD4^+$ T cells. **K** As in (**J**), for insulin-reactive Tregs. Error bars in (**B-K**) represent s.e.m. Data in (**B**) represent n = 5, n = 4, and n = 3 samples for peptide-NB and peptide, blank-NB, and HEL-NB, respectively. One sample represents cells from two mice pooled together. Data in (**C**) represent n = 5, n = 4, and n = 3 samples for peptide-NB, blank-NB and peptide, and HEL-NB, respectively. Data in (**D-G, J-K**) represent n = 6 and n = 5 samples for peptide-NB, and blank-NB and peptide, respectively. Data in (**H**) represent n = 3 samples. Data in (**I**) represent n = 6, n = 3, and n = 5 samples for peptide-NB, HEL-NB, and blank-NB and peptide, respectively. $^*p < 0.05$. (**B**) $p = 0.0289$ comparing peptide-NB and peptide (ANOVA).

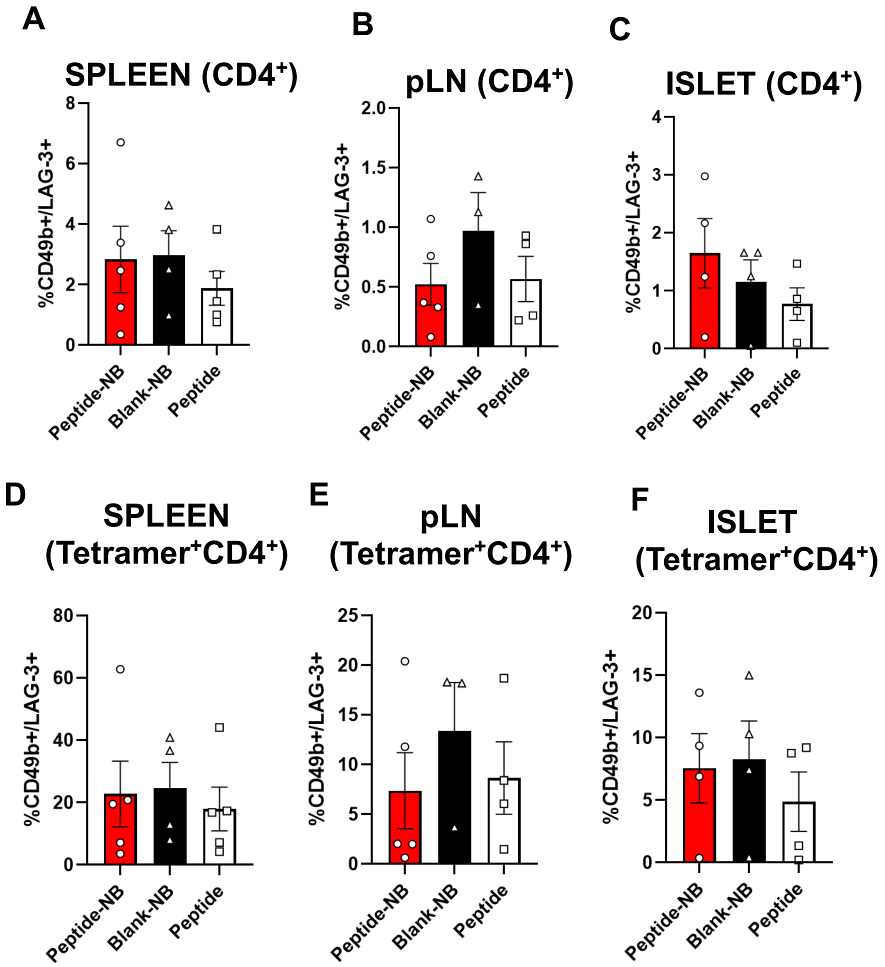

Figure S11. Effect of insulin-peptide-nanobubble treatment in NOD mice on anti-inflammatory $CD4^+CD49b^+LAG\text{-}3^+$ T_R1 cells. A-C Proportion of $LAG\text{-}3^+CD49b^+$ cells amongst all $CD4^+$ T cells in 9 w old NOD mice that had been treated with peptide-nanobubbles, blank nanobubbles, or peptide in (**A**) spleen, (**B**) pancreatic lymph nodes, and (**C**) islets. **D-F** As in (**A-C**), for $LAG\text{-}3^+CD49b^+$ cells amongst insulin-reactive CD4+ T cells. Data in (**A, D**) represent n = 5 samples for peptide-NB and peptide and n = 4 samples for blank-NB. Data in (**B, E**) represent n = 5, n = 4, and n = 3 samples for peptide-NB, and blank-NB, respectively. Data in (**C, F**) represent n = 4 samples. One sample represents cells from two mice pooled together.

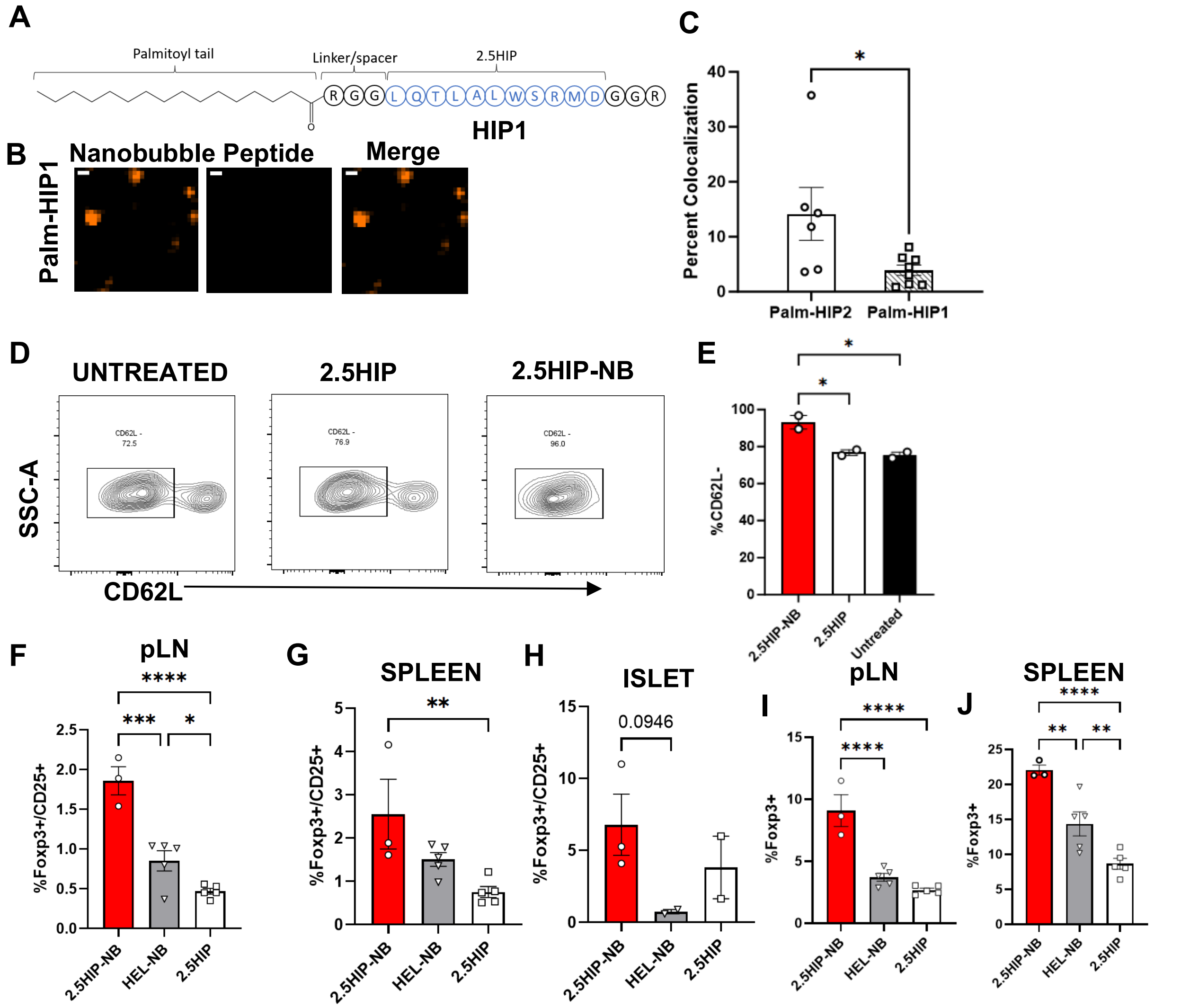

Figure S12. Impact of 2.5HIP linker on nanobubble incorporation and effects of 2.5HIP-NB treatment on lymphoid tissues. **A** Schematic illustrating the design of a palmitoylated 2.5HIP ("HIP1"), where the linker region consists of "RGG" rather than "KKGGCG." **B** Representative confocal images of rhodamine-nanobubbles (orange) containing FITC-labeled palmitoylated HIP1 (green). **C** Percent colocalization between rhodamine and FITC in nanobubbles containing either HIP1, or the HIP2, the palmitoylated 2.5HIP used for experiments (Fig. 5). **D** Representative flow cytometry gating for the identification of CD62L^{-} cells. **E** Percentage of CD62L^{-} cells amongst CD4^{+}CD44^{+} 2.5HIP-reactive splenocytes from NOD mice treated with HIP2-NBs. **F** Proportion of Foxp3^{+}CD25^{+} regulatory T cells amongst CD4^{+} T cells from pancreatic lymph nodes of NOD-SCID mice that had received treatment following adoptive transfer of activated BDC2.5-TCR CD3^{+} T cells. **G** As in (**F**), for cells from the spleen. **H** As in (**F**), for cells from pancreatic islets. **I** As in (**F**), for proportion of Foxp3^{+} cells. **J** As in (**G**), for proportion of Foxp3^{+} cells. Error bars in (**C, E-J**) represent s.e.m. Data in (**C**) represent n = 6 and n = 8 lipid solutions for HIP2 and HIP1, respectively. Data in (**E**) represent n = 2 NOD mice. Data in (**F-G, I-J**) represent n = 3 2.5HIP-NB treated mice and n = 5 HEL-NB and 2.5HIP treated mice. Data in (**H**) represent n = 3 2.5HIP-NB treated mice and 2 HEL-NB and 2.5HIP treated samples, where a sample contains cells pooled from 2-3 mice. Scale bars in (**C**) represent 500 nm. *$p < 0.05$, **$p < 0.01$, ***$p < 0.001$, ****$p < 0.0001$. (**C**) $p = 0.032$ comparing groups indicated (t test, two-tailed). (**E**) $p = 0.0182$ comparing

2.5HIP-NB and 2.5HIP and p = 0.0147 comparing 2.5HIP-NB and no treatment (ANOVA). (**F**) p = 0.0001, < 0.0001, and 0.0265 comparing 2.5HIP-NB and HEL-NB, 2.5HIP-NB and 2.5HIP, and HEL-NB and 2.5HIP, respectively (ANOVA). (**G**) p = 0.005 comparing 2.5HIP-NB and 2.5HIP (ANOVA). (**H**) P = 0.0946 comparing 2.5HIP-NB and HEL-NB (ANOVA). (**I**) p < 0.0001 and <0.0001 comparing 2.5HIP-NB and HEL-NB and 2.5HIP-NB and 2.5HIP, respectively (ANOVA). (**J**) p = 0.003, p < 0.0001 , and p = 0.0076 comparing 2.5HIP-NB and HEL-NB, 2.5HIP-NB and 2.5HIP, and HEL-NB and 2.5HIP, respectively (ANOVA).

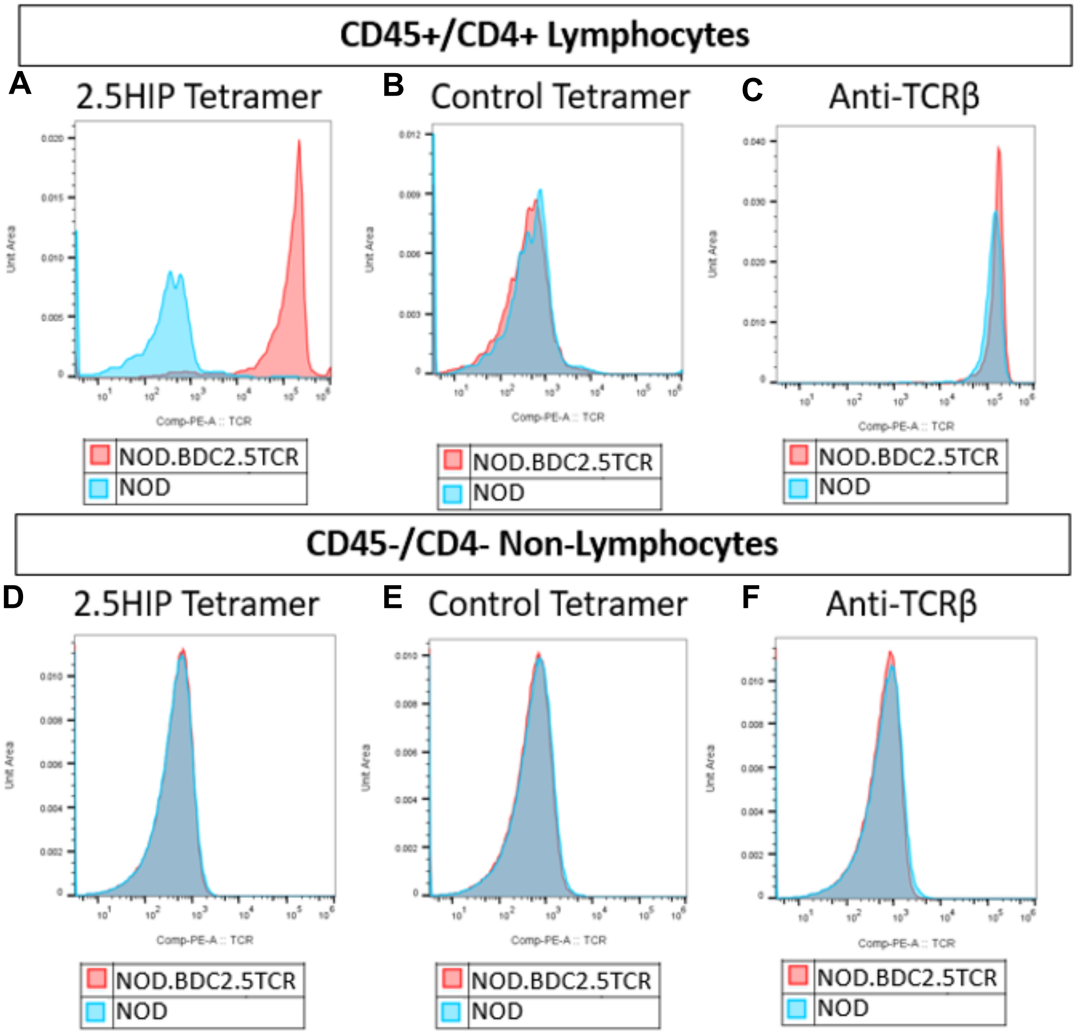

Figure S13. Validation of 2.5HIP MHC tetramer binding specificity using splenocytes. A Histogram of signal intensity from NOD and NOD-BDC2.5TCR-Tg $CD45^{+}CD4^{+}$ splenocytes (T cells) stained with a PE-labeled MHC tetramer specific to 2.5HIP-reactive T-cell receptors (TCRs). **B** As in (**A**), with a control tetramer containing an irrelevant peptide. **C** As in (**A**), with cells stained with PE-labeled anti-TCRβ antibody. (**D-F**) As in (**A-C**), with $CD45^{-}CD4^{-}$ splenocytes (non-immune cells).

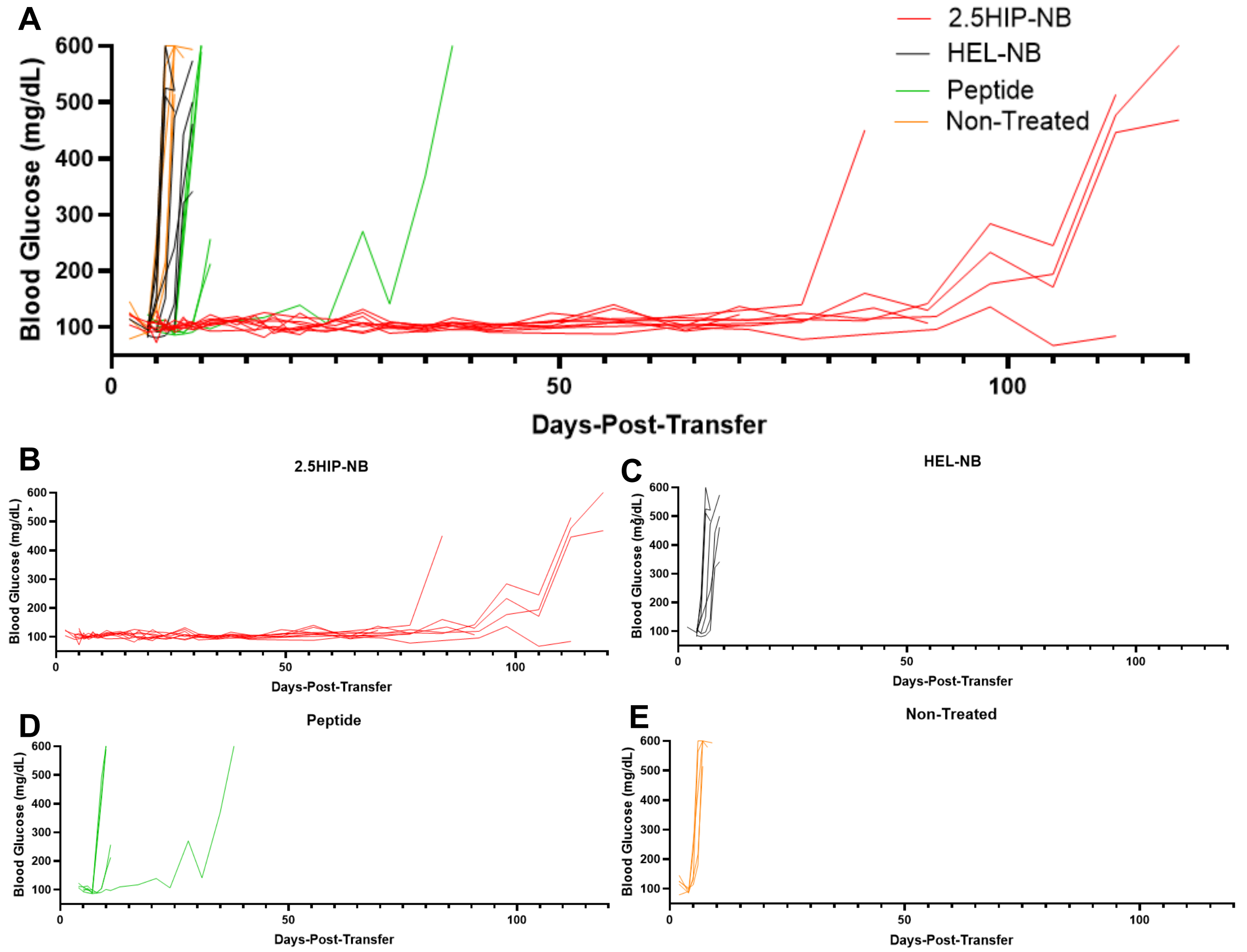

Figure S14. Blood glucose profiles for individual BDC2.5 adoptive transfer mice. Blood glucose over time of BDC2.5 adoptive transfer mice mice (**A**) in all groups and only those administered (**B**) 2.5HIP-NBs (red), (**C**) HEL-NBs (black), (**D**) 2.5HIP (green), or (**E**) non-treated (orange).

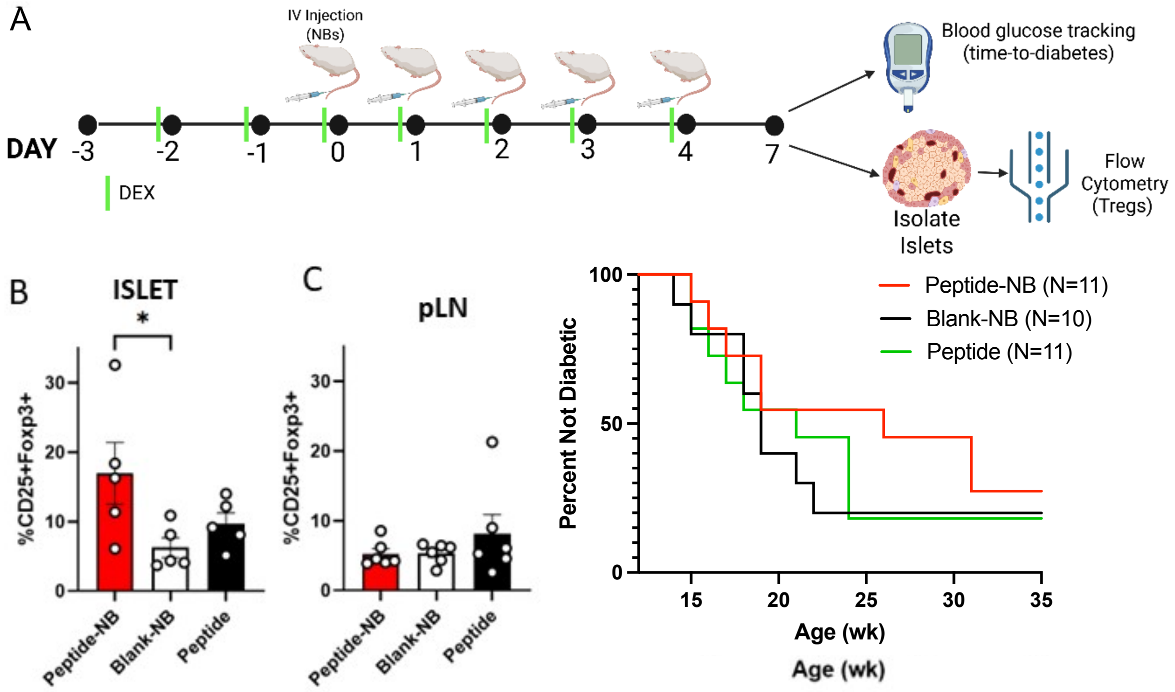

Figure S15. Multi-dose co-administration of insulin peptide-nanobubbles and dexamethasone prevents or delays diabetes onset in NOD mice. A Schematic illustrating protocol for multi-dose co-administration of peptide-nanobubbles and dexamethasone (DEX). **B** Proportion of $Foxp3^{+}CD25^{+}$ Tregs amongst insulin-reactive $CD4^{+}$ T cells in islets of 8 w old NOD mice that had been treated with peptide-nanobubbles, blank-nanobubbles, or peptide. **C** As in (**B**), for pancreatic lymph nodes. d Kaplan-meier curve indicating percentage of NOD mice remaining non-diabetic for 6 w old NOD mice treated with peptide-nanobubbles, blank-nanobubbles, or peptide. Error bars in (**B, C**) represent s.e.m. Data in (**B**) represent n = 6 samples for all groups indicated. Data in (**C**) represent n = 7 samples for all groups indicated. $*p < 0.05$. (**B**) p = 0.0205 comparing peptide-NB and blank-NB (ANOVA).

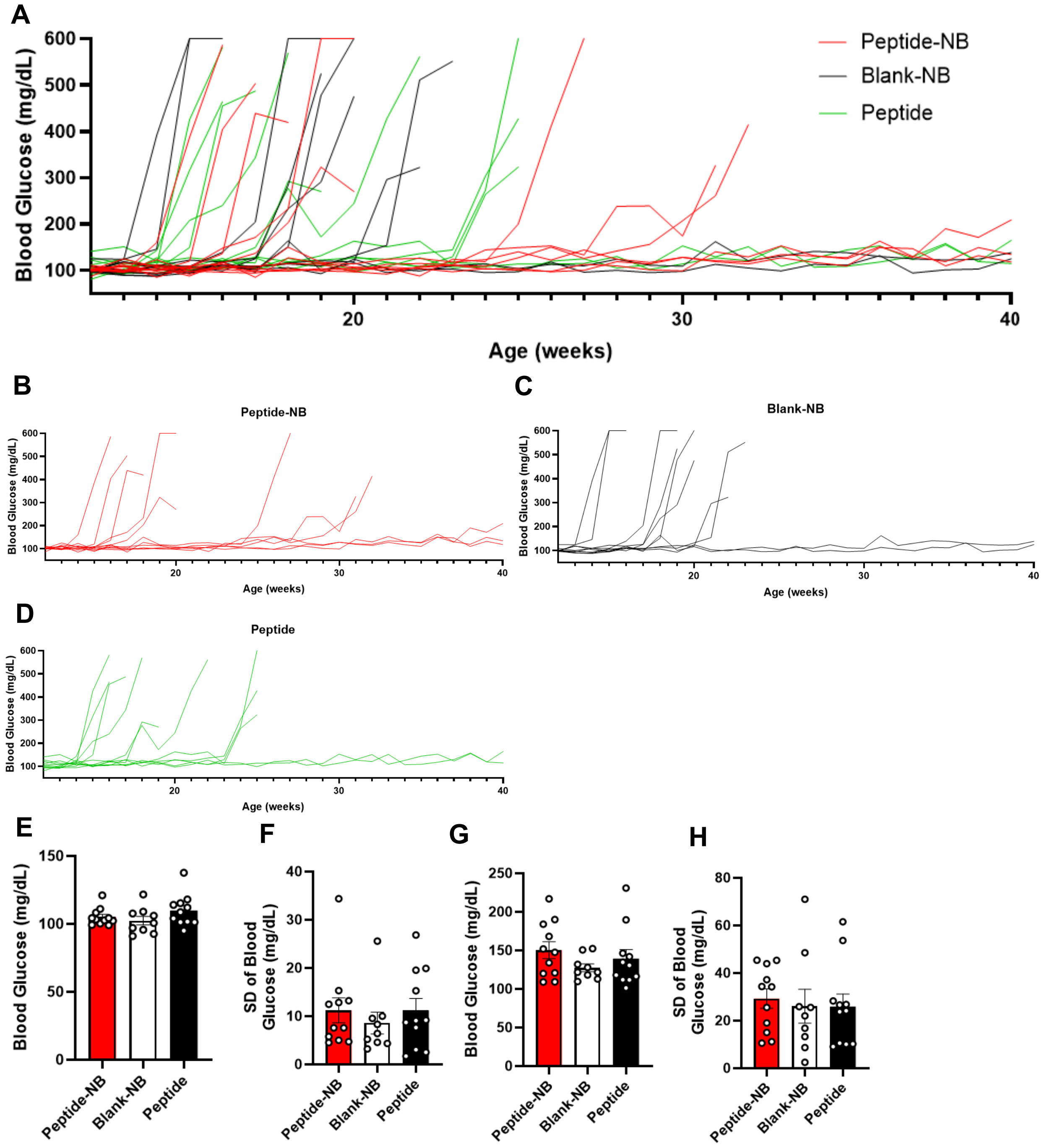

Figure S16. Blood glucose profiles for individual NOD mice. Blood glucose over time of dexamethasone-treated NOD mice (**A**) in all groups and only those administered (**B**) 2.5HIP-NBs (red), (**C**) HEL-NBs (black), or (**D**) 2.5HIP (green). (**E-H**) Average (**E, G**) and standard deviation (**F, H**) of blood glucose amongst mice at ages 12-14 w (**E, F**) and at the 3 w immediatley preceding diabetes onset, or at weeks 38-40 if non-diabetic (**G, H**). Data in (**E-H**) represent N = 11, 9, and 11 mice for peptide-NB, blank-NB, and peptide, respecitvely.
